# UniHAR: A unified hierarchical transformer with multi-scale temporal modeling for wearable human activity recognition

**DOI:** 10.1371/journal.pone.0359774

**Published:** 2026-10-30

**Authors:** Memoona, Muhammad Haseeb Hashir, Sung Won Kim

**Affiliations:** 1 Department of Information and Communication Engineering, Yeungnam University, Gyeongsan, Gyeongbuk, Republic of Korea; 2 Department of Information and Communication Engineering, Yeungnam University, Gyeongsan, Gyeongbuk, Republic of Korea; 3 School of Computer Science and Engineering, Yeungnam University, Gyeongsan, Gyeongbuk, Republic of Korea; Air University, PAKISTAN

## Abstract

UniHAR is a lightweight hierarchical multi-scale transformer based on wearable sensor data that has to be able to model time well; be able to recognize human activity across different datasets; and do so in real-time. There are several problems with what currently exists. UniHAR uses four major components: universal channel harmonization; adaptive multi-scale patch embedding; scale-adaptive Fourier cross-attention; factorized temporal-channel attention. The components allow for the ability to use various sensors and enables the low-complexity multi-scale fusion of features. UniHAR achieved an accuracy of 96.35%; 94.45%; 96.44%; and 99.23% on UCI-HAR, Opportunity, Daphnet, and PAMAP2 datasets, while being very lightweight and having high-speed inference capabilities; therefore, UniHAR is suitable for real-time healthcare applications.

## 1 Introduction

Human activity recognition (HAR), is a method to automatically classify the type of activity that people are undertaking by analyzing data gathered from sensors through machine learning and signal processing [1,2]. The most common areas where this can be used are: medical monitoring [3–5], athletic analysis [6,7], fitness optimization [8–14], surveillance [15], and human-computer interaction [16]. There are two types of methods for HAR vision based and sensor based [16–19]; Both vision-based methods will gather information from optical streams [19], Sensor-based methods will utilize accelerometer, gyroscope and orientation sensors [19,20], and offer greater portability than vision systems [20].. Yet growing deep network complexity imposes prohibitive computational and memory costs in mobile and embedded settings [21,22], motivating lightweight architectures that preserve recognition accuracy [22].

Beyond this general efficiency concern, sensor-based HAR presents four domain-specific challenges that, taken together, motivate the design of UniHAR. First, HAR benchmarks differ widely in *sensor configuration*, so architectures built around a fixed channel-dependent weight matrix cannot be transferred across datasets without redesigning the input layer. Second, human motion is inherently *multi-scale*: fine-grained wrist or hand gestures coexist with coarse, whole-body postural transitions within the same activity window, so a model built around a single fixed temporal resolution systematically under-represents one class of motion at the expense of the other. Third, wearable signals such as gait cycles, pedaling cadence, and repetitive exercise are strongly *periodic*, a structure that standard time-domain self-attention does not exploit directly and must instead learn implicitly from data. Fourth, because HAR models are frequently intended for deployment on mobile or embedded devices, jointly modeling temporal dynamics and feature-channel dependencies must be done without incurring the quadratic cost of full self-attention, or the efficiency gains motivated above are lost in practice.

To address these four challenges jointly, we propose **UniHAR**, a hierarchical multi-scale Transformer built around four components, applied in sequence and each targeting one of the challenges described above: (i) **Universal Channel Harmonization (UCH)** projects heterogeneous sensor layouts into a unified, fixed-dimensional feature space using channel-count-agnostic per-channel descriptors, enabling cross-dataset generalization without dataset-specific redesign; (ii) **Adaptive Multi-Scale Patch Embedding (AMSPE)** encodes sequences at fine, medium, and coarse temporal resolutions whose patch sizes are tied to the physical sampling rate, so that fine- and coarse-grained motion are each represented at an appropriate scale rather than being forced into a single resolution; (iii) **Scale-Adaptive Fourier Cross-Attention (SAFCA)** combines FFT-based intra-scale mixing, which directly captures the periodic structure of wearable signals, with cross-scale attention that propagates context across the three temporal resolutions; and (iv) **Factorized Temporal-Channel Attention (FTCA)** factorizes attention across the temporal (token) dimension and the feature-channel (embedding) dimension, avoiding the quadratic cost of joint attention and yielding a form suitable for real-time, edge-deployed inference. Together, these components allow a single set of parameters to be trained once and applied across datasets with different channel counts, sampling rates, and activity taxonomies, without being re-initialized or re-trained per dataset. On UCI-HAR, Opportunity, Daphnet, and PAMAP2, UniHAR achieves 96.35%, 94.45%, 96.44%, and 99.23% accuracy within a 0.7–0.9M parameter footprint.

The contributions are:

- **UniHAR**: a hierarchical multi-scale Transformer for fine- and coarse-grained motion recognition, addressing the multi-scale nature of human activity.
- **Universal Channel Harmonization (UCH)**: a channel-agnostic projection enabling generalization across heterogeneous sensor configurations without architectural modification.
- **Efficient attention** via Scale-Adaptive Fourier Cross-Attention (SAFCA) and Factorized Temporal-Channel Attention (FTCA) for scalable multi-scale fusion at reduced complexity, addressing signal periodicity and the computational constraints of edge deployment.
- **State-of-the-art results** on four benchmarks with ablation and efficiency analysis.
- **Frequency-domain insights** showing Fourier processing is especially effective for periodic activities.

## 2 Related work

### 2.1 Traditional and deep learning approaches for wearable HAR

Early HAR based on sensors was primarily dependent on extracting hand-crafted statistical and frequency characteristics (e.g., mean, variance; FFT Coefficients; Zero-Cross Rate) from a fixed sliding window and classifying with traditional machine learning models (Decision Trees, SVM etc.) [23]. While these methods were relatively easy to interpret, they required domain knowledge for feature extraction and had poor generalization properties over sensor configurations.

The application of deep learning eliminated the need to manually design features by hand. Yang et al. [24] showed that a CNN operating directly on a multichannel raw signal time series could beat baseline systems engineered using pre-designed features on the UCI-HAR database. Ordóñez and Roggen [25] employed both convolutional and bidirectional LSTM layers in a Deep Convolutional Long Short Term Memory (CNN-LSTM) model which learned joint spatial (inter sensor) and temporal information on the opportunity data set significantly increasing accuracy. The authors of Hammerla et al. [26], in their comparative study of deep, convolutional, and recurrent neural network models on four HAR benchmark data sets, showed that LSTM-based models were the most accurate consistently among all architectures tested. However, they also found that no model was best suited to all of the individual data sets; therefore, there is still an unmet need for universal (unified) design approaches. Subsequently, Guan et al. [27] expanded upon the use of recurrent models as ensembles as well as using residual formulations of these models which produced increased accuracy with reduced variability during model training. Recent InceptionTime-Style Models [28], which utilize Multi-Scale Convolutions Over Time-Series Data (to find patterns at varying levels of granularity), have been able to perform well by utilizing the various scales within one Convolution Block. Additionally, Ronald et al. [29], utilized iSPLInception (a variant of InceptionResNet) for performing strong results on the four benchmark datasets that were used in this study.

These advancements notwithstanding, there are fundamental limitations in both pure convolutional and Recurrent models. The first limitation is that using Local Receptive Fields with Fixed Length Kernels does not provide an efficient method of finding Long-Range Temporal Dependencies unless they are stacked very deeply. Secondly, each model type utilizes Dataset Specific Input Layers; therefore, if the Channel Count (*C*) changes among deployments, training or Structural Modifications would be required.

Beyond this general efficiency concern, a growing body of work targets the inference cost of deep HAR models directly rather than relying on generic complexity arguments [30]. proposed Rep-LOM, which fuses 1×1 convolutions with structural reparameterization and decoupled fully-connected (DFC) attention, collapsing a multi-branch training-time topology into a single-branch inference-time network to reduce FLOPs and parameters without sacrificing accuracy. Extending this direction [31], introduced Rep-MMB, which bridges mobile CNN and Transformer design to further improve the accuracy-efficiency trade-off of sensor-based HAR on resource-constrained hardware. Rather than statically shrinking the network [32], addressed inference cost through dynamic early-exit: a meta-learned sample-reweighting scheme aligns training-time loss weighting with the test-time behavior of multi-exit classifiers, letting easy activity samples exit early while harder samples are routed to deeper classifiers, which consistently improves the accuracy-cost trade-off over conventional early-exit training. These works illustrate that recent progress in efficient HAR increasingly relies on architectural reparameterization and adaptive inference rather than on hand-designed lightweight blocks alone; UniHAR’s FTCA and SAFCA modules target the same computational bottleneck through a complementary sequence-factorization strategy rather than reparameterization or early exiting.

A parallel line of work sidesteps direct sequence modeling by first transforming the sensor time-series into a 2D image representation and then applying image-domain CNNs. These representations include time-dependent direction/position images [33], cross-modal fusions of Markov transition fields, recurrence plots, and Gramian angular fields [34], relative position images derived from spatially distributed flexible sensor arrays [35], and wavelet scalograms combined with texture descriptors [36], with reported accuracies exceeding 99% on constrained gymnastics recognition using a dedicated 11-node sensor network [37]. These approaches show that engineered image transformations can substantially improve feature separability within a single, narrowly scoped activity domain. However, they are evaluated on task-specific rigs with fixed node counts and placements (e.g., an 11-node gymnastics network or a 16-channel lower-limb array) rather than the heterogeneous, cross-dataset channel counts addressed by UCH, and they rely on an explicit image-transformation preprocessing stage rather than operating on the raw multichannel sequence. UniHAR instead retains the sequence representation throughout, applying multi-scale tokenization and frequency-domain mixing directly to the raw signal without an intermediate image-transformation step.

### 2.2 Transformer models for time-series and HAR

The Transformer [38], based on multi-head self-attention, replaced recurrence with direct pairwise token interactions and became the dominant architecture in natural language processing. Adapting this mechanism to time-series data requires addressing the quadratic cost of attention over long sequences and the lack of positional inductive bias.

Zerveas et al. [39] introduced a Transformer-based framework for multivariate time-series representation learning, demonstrating that pre-training on masked inputs and fine-tuning on downstream tasks improves classification and regression performance substantially over supervised-only baselines. For long-sequence forecasting, Zhou et al. [40] proposed Informer, which replaces full self-attention with a sparse ProbSparse mechanism to achieve O(NlogN) complexity, and Wu et al. [41] introduced Autoformer, which replaces attention with an auto-correlation mechanism over decomposed seasonal–trend components. Nie et al. [42] showed that segmenting time-series into non-overlapping patches before applying Transformer attention significantly reduces sequence length and improves long-range forecasting, a patching idea that directly motivates the multi-scale patch tokenization in AMSPE.

In the HAR domain specifically, Xu et al. [43] proposed LIMU-BERT, which pre-trains a BERT-style Transformer on raw IMU data with masked sensor modeling, achieving strong generalization across HAR benchmarks with limited labeled data. Tang et al. [44] proposed a multi-head self-attention module for activity recognition that weighs temporal segments adaptively, showing that attention-based pooling outperforms global average pooling for IMU sequences.

A parallel and more recent trend addresses HAR data scarcity and cross-dataset heterogeneity by adapting large pretrained Time-Series Foundation Models (TSFMs) rather than training HAR-specific backbones from scratch [45]. proposed DeepSenseMoE, a multi-scale convolutional Mixture-of-Experts module for parameter-efficient fine-tuning of general-purpose TSFMs on few-shot wearable activity recognition, unlocking general time-series representations for HAR despite severe annotation scarcity and sensor heterogeneity. In a related direction [46], introduced Sensor-Prompt Tuning, which aligns TSFMs with motion-sensor inputs through lightweight prompt-based adaptation for few-shot activity recognition, avoiding full fine-tuning of the foundation model. Both works reflect a paradigm shift toward reusing large-scale pretrained time-series representations for HAR, in contrast to UniHAR’s from-scratch, channel-agnostic harmonization strategy (UCH), which addresses the same cross-dataset heterogeneity problem without relying on external large-scale pretraining.

Common to all of these works is a fixed-channel input assumption and the use of a single temporal resolution. UniHAR extends this line by operating simultaneously at three temporal scales and by introducing a channel-agnostic harmonization module that removes the fixed-channel constraint without dataset-specific redesign.

### 2.3 Multi-scale and hierarchical architectures

Human motion is inherently multi-scale: fine-grained wrist movements and coarse whole-body postural transitions coexist within a single activity window. Capturing this structure requires representations at multiple temporal granularities.

In computer vision, Liu et al. [47] introduced the Swin Transformer, which processes image patches in a hierarchical, shifted-window attention scheme, producing features at progressively coarser spatial resolutions. Fan et al. [48] extended this with Multiscale Vision Transformers (MViT), which pool spatial and channel dimensions across stages to model visual content at multiple scales. These hierarchical designs demonstrate that scale-aware representation learning improves recognition performance on structured signals.

For time-series, InceptionTime [28] applies parallel convolutions with kernels of different lengths before concatenating the resulting feature maps, capturing temporal patterns at multiple granularities within each layer. However, this approach applies fixed kernel sizes independent of the sampling rate and does not perform cross-scale information fusion across encoder layers.

UniHAR generalizes multi-scale design to wearable HAR by: (i) adapting patch sizes to the physical sampling rate rather than fixing them as hyperparameters, (ii) explicitly fusing information across scales at every encoder block via SAFCA and ISIE, and (iii) learning scale-specific attention weights at classification time rather than concatenating all scale outputs uniformly.

### 2.4 Frequency-domain methods for sequence modeling

Wearable sensor signals exhibit strong periodic structure–-gait cycles, pedaling cadence, repetitive exercise–-that is naturally represented in the frequency domain. Several recent works have incorporated Fourier processing into sequence models to exploit this structure.

Lee et al. [49] replaced multi-head self-attention in standard Transformers with unlearned Fourier transforms applied over both the sequence and embedding dimensions, achieving near-BERT-level accuracy on NLP benchmarks at a fraction of the computational cost. Zhou et al. [50] proposed FEDformer, which applies Fourier and wavelet transforms to decompose time-series into frequency components before attention, improving long-term forecasting while reducing complexity. Wu et al. [41] also leverage frequency-domain structure through their Auto-Correlation mechanism, which identifies recurrent dependencies by computing the FFT of the time-delay similarity function.

These works primarily target forecasting and NLP, not multi-sensor HAR, and do not combine frequency-domain intra-scale mixing with cross-scale attention. SAFCA in UniHAR unifies both: FFT-based token mixing captures periodic patterns within each scale, while cross-scale attention propagates context across temporal resolutions in a single efficient block.

### 2.5 Efficient attention mechanisms for long sequences

Standard self-attention scales as *O*(*N*^2^) in sequence length, motivating approximations for long-sequence inputs. Katharopoulos et al. [51] reformulated dot-product attention using feature-map kernel approximations, showing that the cumulative key-value product can be maintained in linear time and constant memory, yielding *O*(*N*) complexity. This linear attention formulation is adopted in UniHAR’s FTCA temporal attention sub-layer to keep per-token costs tractable.

### 2.6 Cross-dataset generalization in HAR

Cross-dataset generalization has received comparatively little attention in HAR. Most published models are evaluated on a single benchmark and assume fixed channel counts at training and test time. Wilson et al. [52] studied domain adaptation between HAR datasets with different sensor placements, demonstrating that naive fine-tuning degrades significantly without explicit alignment mechanisms. Haresamudram et al. [53] proposed contrastive predictive coding on unlabeled IMU sequences to learn transferable representations, reducing the gap between source and target distributions. Nevertheless, these approaches still require the input channel count to be identical between domains, or resort to channel selection/alignment steps that discard sensor channels not present in the target.

UniHAR addresses cross-dataset generalization at the architecture level through UCH, which learns a shared per-channel scorer that maps variable-length sensor inputs to a fixed-dimensional representation without dataset-specific re-training. To our knowledge, UniHAR is the first wearable HAR architecture designed to jointly address multi-scale temporal modeling, heterogeneous channel harmonization, and efficient edge deployment within a single unified framework.

## 3 Methodology

This section presents *UniHAR*, a single end-to-end trainable framework for wearable sensor-based HAR that operates across datasets with varying channel counts, sampling rates, and activity taxonomies without dataset-specific backbone modifications. The pipeline proceeds through four stages: (i) Universal Channel Harmonization (UCH), (ii) Adaptive Multi-Scale Patch Embedding (AMSPE), (iii) a Hierarchical Transformer Encoder (SAFCA–ISIE–FTCA blocks), and (iv) Multi-Scale Aggregation with Adaptive Classification. The overall architecture is illustrated in Fig 1.

**Fig 1:**
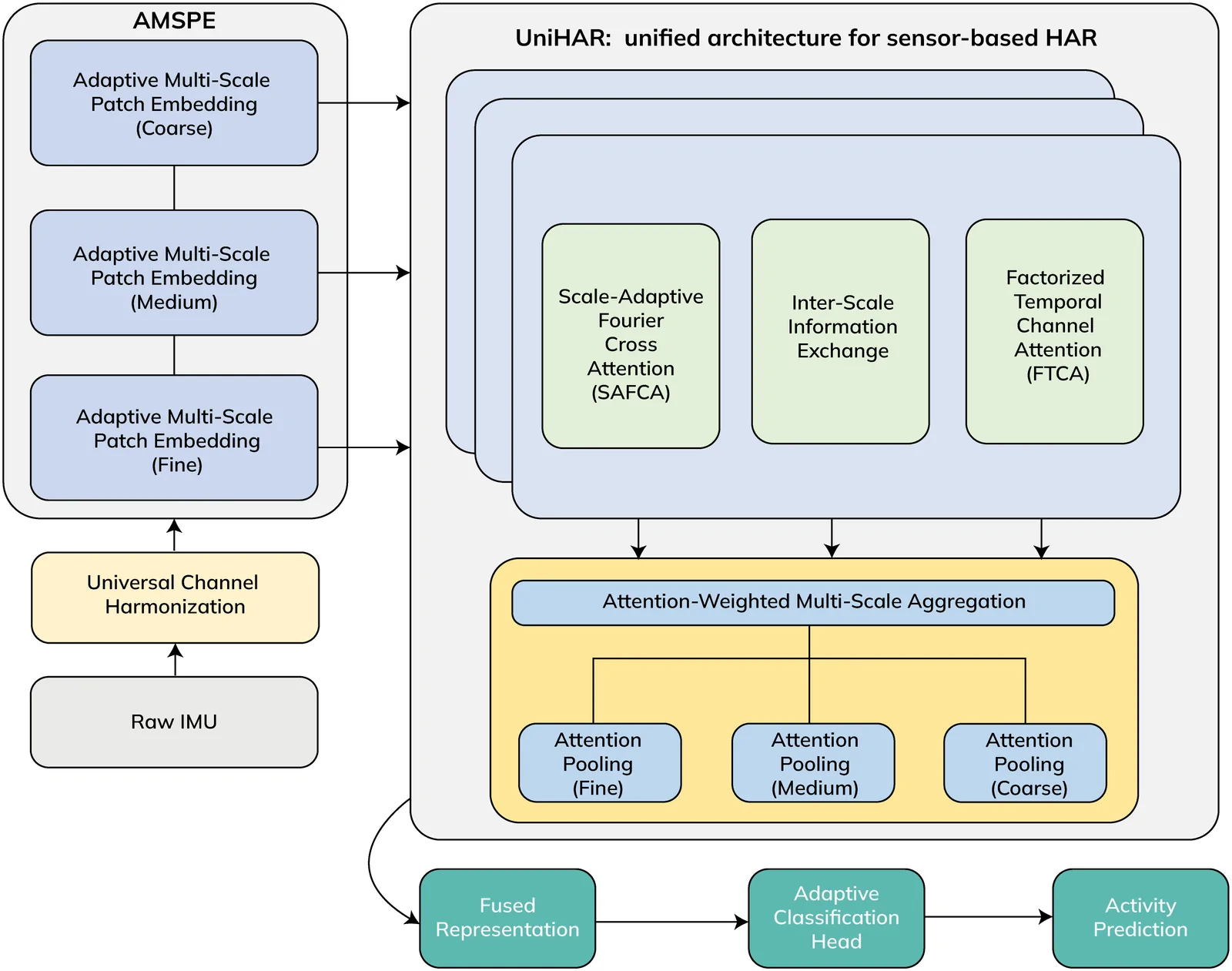
Overall architecture of the proposed framework.

### 3.1 Problem formulation

Let $𝐗\in {\mathbb{R}}^{T\times C}$ be a windowed wearable sensor segment with *T* time-steps, *C* channels, and label y∈{1,...,K}. Given $𝒟=\{({𝐗}_{i},y_{i}){\}}_{i=1}^{N}$, subject-independent splits evaluate cross-subject generalization. UniHAR must simultaneously accommodate three sources of inter-dataset heterogeneity: (i) temporal (*T*, $f_{s}$), (ii) sensory (*C*, ranging 9/113/9/18 across our benchmarks), and (iii) task (*K*, binary to 18-class). The objective is to learn


$$\begin{array}{c} f_{\theta }:{\mathbb{R}}^{T\times C}⟶{\mathbb{R}}^{K} \end{array}$$ (1)

whose parameters θ are not re-initialized or re-trained per dataset.

### 3.2 Universal channel harmonization (UCH)

Directly sharing a weight matrix $𝐖\in {\mathbb{R}}^{C\times d}$ across datasets with different channel counts *C* is impossible without modification. UCH resolves this by replacing the channel-count-dependent weight with a *shared per-channel scorer* that operates on a fixed-size descriptor computed independently for each channel, regardless of how many channels *C* the input contains.

#### 3.2.1 Per-channel descriptor extraction.

For each channel c∈{1,...,C}, a fixed-length descriptor $\psi_{c}\in {\mathbb{R}}^{d_{\psi }}$ is computed from the channel’s time-series ${𝐱}_{c}\in {\mathbb{R}}^{T}$ using a compact set of statistics that are well-defined for any *T*:


$$\begin{array}{c} \psi_{c}={\left[\mu_{c},\;\sigma_{c},\;\gamma_{1,c},\;\gamma_{2,c},\;{𝐩}_{c}^{⊤}\right]}^{⊤}\in {\mathbb{R}}^{d_{\psi }}, \end{array}$$ (2)

where $\mu_{c}$, $\sigma_{c}$, $\gamma_{1,c}$, $\gamma_{2,c}$ are the mean, standard deviation, skewness, and excess kurtosis of ${𝐱}_{c}$, and ${𝐩}_{c}\in {\mathbb{R}}^{d_{\psi }-4}$ are the top-$\left(d_{\psi }-4\right)$ normalized spectral power bins of ${𝐱}_{c}$. We set $d_{\psi }=16$ in all experiments. We adopt statistical descriptors rather than a learned encoder to ensure robustness across heterogeneous sensor modalities without introducing dataset-specific feature extractors; the statistics are well-defined for any sensor type and require no pre-training. The descriptor is channel-count agnostic: the same $d_{\psi }$-dimensional vector is produced for any channel in any dataset.

#### 3.2.2 Shared group-assignment scorer.

A two-layer MLP with parameters shared across all datasets and all channels maps each descriptor to a soft group-membership vector:


$$\begin{array}{c} {𝐚}_{c}=softmax\;\left({MLP}_{g}\;\left(\psi_{c}\right)\right)\in {\mathbb{R}}^{M}, \end{array}$$ (3)

where ${MLP}_{g}:{\mathbb{R}}^{d_{\psi }}\to {\mathbb{R}}^{M}$ has fixed input and output dimensions regardless of *C*. Stacking all *C* channel assignments gives $𝐀=[{𝐚}_{1},...,{𝐚}_{C}{]}^{⊤}\in {\mathbb{R}}^{C\times M}$. Because the same MLP weights are used for every channel in every dataset, UCH shares all parameters across datasets and adapts only through the input descriptors, which vary with the actual sensor data.

#### 3.2.3 Soft-weighted group projection.

Each group embedding is a soft weighted sum over all channels:


$$\begin{array}{c} {\hat{𝐗}}_{m}=𝐗\;diag({𝐀}_{:,m})\in {\mathbb{R}}^{T\times C},\;m=1,...,M, \end{array}$$ (4)

followed by a 1×1 Conv1D projection to *d*/*M* features:


$$\begin{array}{c} {𝐇}_{m}=Conv1D\;\left({\hat{𝐗}}_{m};\;out=d/M\right)\in {\mathbb{R}}^{T\times d/M}. \end{array}$$ (5)

#### 3.2.4 Fusion.

All group embeddings are concatenated and linearly fused:


$$\begin{array}{c} {𝐗}_{u}=LayerNorm\;\left({𝐖}_{f}\;Concat[{𝐇}_{1},...,{𝐇}_{M}]\right)\in {\mathbb{R}}^{T\times d}, \end{array}$$ (6)

with ${𝐖}_{f}\in {\mathbb{R}}^{d\times d}$. The only parameter that changes shape with *C* is the Conv1D kernel in Eq. (5), which operates on the weighted input ${\hat{𝐗}}_{m}\in {\mathbb{R}}^{T\times C}$ but projects along the channel dimension independently per time-step. In practice, we implement this as a shared 1×1 convolution whose *C*-dimensional input is zero-padded to the maximum *C* seen during training; channels beyond the training maximum are assigned near-zero group weights by the scorer, making the padding effectively inactive. We acknowledge that this padding strategy imposes a soft upper bound on supported channel counts during training; supporting truly unbounded *C* at test time remains an open direction.

### 3.3 Adaptive multi-scale patch embedding (AMSPE)

Direct self-attention over raw time-steps costs *O*(*T*^2^) and treats all temporal granularities equally. AMSPE constructs three token sequences whose patch sizes are tied to the physical sampling rate, ensuring each scale corresponds to a meaningful motion time-scale.

#### 3.3.1 Target-duration patch computation.

For a given sampling rate $f_{s}$, the patch size for scale s∈{f,m,c} is determined by a target patch duration $\tau_{s}$ in seconds:


$$\begin{array}{c} p_{f}=\max\;\left(4,\;\lfloor \tau_{f}f_{s}\rfloor \right),\;p_{m}=\max\;\left(8,\;\lfloor \tau_{m}f_{s}\rfloor \right),\;p_{c}=\max\;\left(16,\;\lfloor \tau_{c}f_{s}\rfloor \right), \end{array}$$ (7)

with $\tau_{f}=0.15$ s, $\tau_{m}=0.35$ s, $\tau_{c}=0.70$ s. For example, at $f_{s}=50$ Hz: $p_{f}=7$, $p_{m}=17$, $p_{c}=35$ time-steps, corresponding to approximately 140 ms, 340 ms, and 700 ms motion windows. Strides are ${str}_{s}=\max(\lfloor p_{s}/2\rfloor ,2)$ (50% overlap). The lower bounds in Eq. (7) prevent degenerate patches at very low sampling rates.

#### 3.3.2 Token generation and encoding.

For each scale *s*, a Conv1D layer produces $N_{s}=\lfloor (T-p_{s})/{str}_{s}\rfloor +1$ tokens:


$$\begin{array}{c} {𝐏}^{s}=Conv1D\;\left({𝐗}_{u};\;kernel=p_{s},\;stride={str}_{s},\;out=d\right)\in {\mathbb{R}}^{N_{s}\times d}. \end{array}$$ (8)

Learnable positional embeddings ${PE}^{s}\in {\mathbb{R}}^{N_{s}\times d}$, per-scale embeddings ${SE}^{s}\in {\mathbb{R}}^{1\times d}$ (broadcast), and a global temporal summary $TE\in {\mathbb{R}}^{1\times d}$ (mean of ${𝐗}_{u}$ projected to *d*) are summed:


$$\begin{array}{c} {𝐙}^{s}={𝐏}^{s}+{PE}^{s}+{SE}^{s}+TE,\;s\in \{f,m,c\}, \end{array}$$ (9)

giving $N_{f}>N_{m}>N_{c}$ tokens, all of dimension *d*.

### 3.4 Hierarchical transformer encoder

The encoder comprises *L* = 4 identical blocks, each processing $\left\{{𝐙}^{f},{𝐙}^{m},{𝐙}^{c}\right\}$ through SAFCA, ISIE, and FTCA sub-layers with residual connections and LayerNorm after each sub-layer.

#### 3.4.1 Scale-adaptive fourier cross-attention (SAFCA).

**Step 1: Fourier Intra-Scale Mixing.** A 1D FFT is applied along the token dimension ($N_{s}$ tokens, each a row vector in ${\mathbb{R}}^{d}$), independently per feature channel:


$$\begin{array}{c} {𝐅}^{s}={ℱ}_{N_{s}}\;\left({𝐙}^{s}\right)\in {\mathbb{C}}^{N_{s}\times d}. \end{array}$$ (10)

Learnable complex-valued spectral filters $\Phi^{s}\in {\mathbb{C}}^{N_{s}\times d}$ modulate each frequency–feature entry element-wise:


$$\begin{array}{c} {\tilde{𝐅}}^{s}=\Phi^{s}⊙{𝐅}^{s}. \end{array}$$ (11)

The inverse FFT is applied and the real part retained before any cross-scale operation (so all subsequent Q, K, V are formed from real-valued signals):


$$\begin{array}{c} {\tilde{𝐙}}^{s}=Re\;\left({ℱ}_{N_{s}}^{-1}\;\left({\tilde{𝐅}}^{s}\right)\right)\in {\mathbb{R}}^{N_{s}\times d}. \end{array}$$ (12)

The pipeline order is: ${𝐙}^{s}\overset{ℱ\;}{\to }{𝐅}^{s}\overset{\Phi^{s}\;}{\to }{\tilde{𝐅}}^{s}\overset{{ℱ}^{-1}\;}{\to }{\tilde{𝐙}}^{s}\to$ cross-scale attention.

**Step 2: Cross-Scale Attention.** Queries, keys, and values are formed from the Fourier-mixed outputs ${\tilde{𝐙}}^{s}$. Because $N_{f},N_{m},N_{c}$ differ, keys and values from source scale $s^{\prime }$ are pooled to the query length before attention:


$$\begin{array}{c} {𝐊}^{s\leftarrow s^{\prime }}={Pool}_{N_{s}}\;\left({\tilde{𝐙}}^{s^{\prime }}{𝐖}_{K}\right),\;{𝐕}^{s\leftarrow s^{\prime }}={Pool}_{N_{s}}\;\left({\tilde{𝐙}}^{s^{\prime }}{𝐖}_{V}\right), \end{array}$$ (13)

$$\begin{array}{c} {𝐂}^{s\leftarrow s^{\prime }}=softmax\;\left(\frac{{\tilde{𝐙}}^{s}{𝐖}_{Q}{\left({𝐊}^{s\leftarrow s^{\prime }}\right)}^{⊤}}{\sqrt{d_{h}}}\right){𝐕}^{s\leftarrow s^{\prime }}, \end{array}$$ (14)

where ${𝐖}_{Q},{𝐖}_{K},{𝐖}_{V}\in {\mathbb{R}}^{d\times d}$ and $d_{h}=d/H$.

**Step 3: Gated Fusion.** Following the row-vector convention (${𝐙}^{s}\in {\mathbb{R}}^{N_{s}\times d}$, tokens as rows), the gate is computed as:


$$\begin{array}{c} {𝐆}^{s}=\sigma \;\left({𝐙}^{s}\;{𝐖}_{g}^{s}\right),\;{𝐖}_{g}^{s}\in {\mathbb{R}}^{d\times d}, \end{array}$$ (15)

yielding ${𝐆}^{s}\in {\mathbb{R}}^{N_{s}\times d}$. The final SAFCA output is:


$$\begin{array}{c} {𝐙}_{SAFCA}^{s}=LayerNorm\;({𝐆}^{s}⊙{\tilde{𝐙}}^{s}+(1-{𝐆}^{s})⊙\frac{1}{2}\sum_{s^{\prime }\neq s}{𝐂}^{s\leftarrow s^{\prime }}+{𝐙}^{s}). \end{array}$$ (16)

All terms are ${\mathbb{R}}^{N_{s}\times d}$; element-wise operations are dimensionally consistent.

Fig 2 shows the schematic illustration of the proposed Scale-Adaptive Fourier Cross-Attention module.

**Fig 2:**
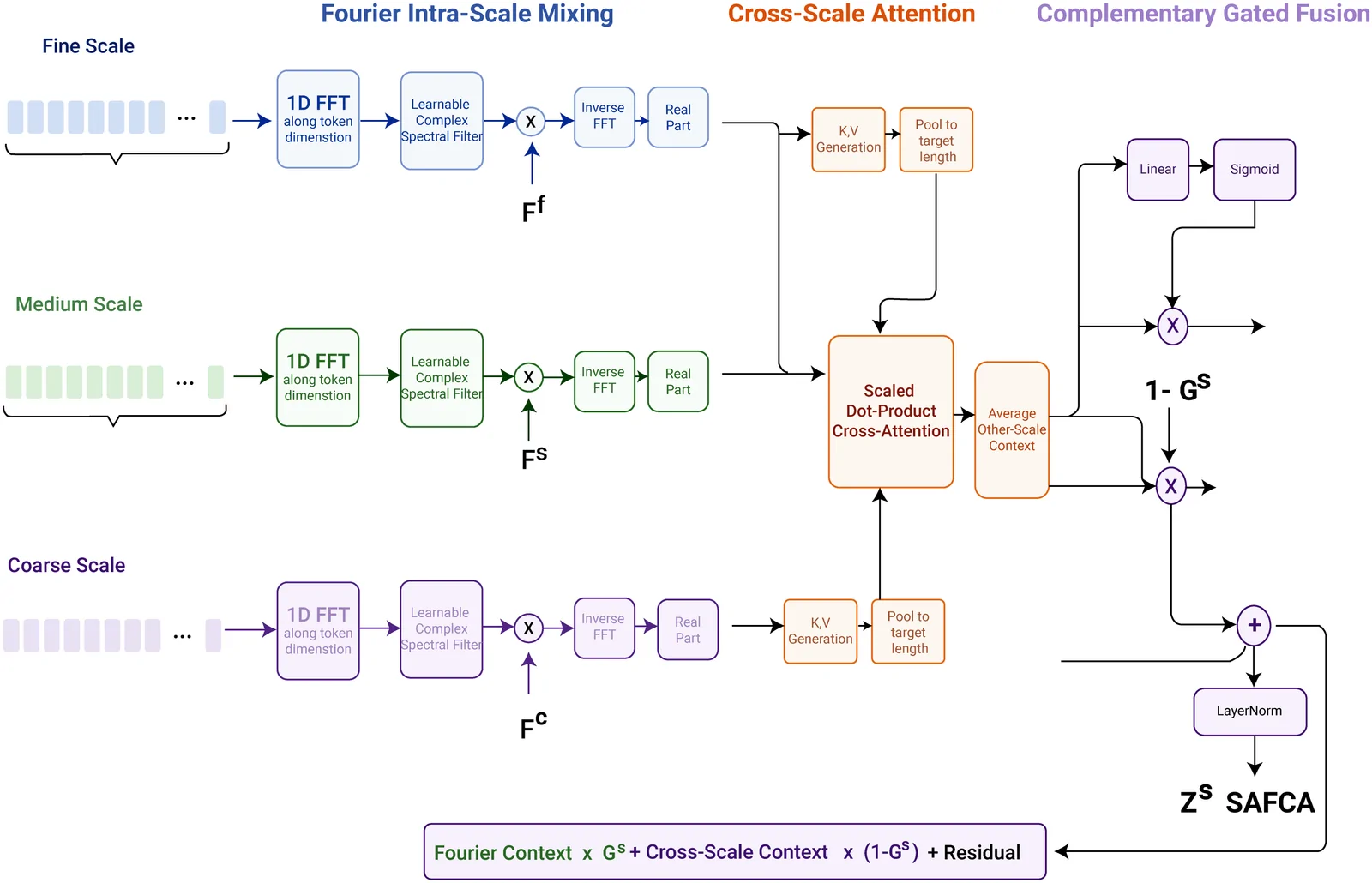
Schematic illustration of the proposed Scale-Adaptive Fourier Cross-Attention (SAFCA) module.

#### 3.4.2 Inter-scale information exchange (ISIE).

SAFCA injects cross-scale context through attention but leaves each scale normalized independently, allowing representations to drift. ISIE enforces explicit alignment through directed message passing in the order coarse→medium→fine.

This direction is chosen for the following reason: the coarse-scale stream summarizes the longest time context and thus carries the most stable global activity state. Propagating this global context down to finer scales grounds local motion features in the overall activity label before fine-grained temporal modeling begins. The reverse direction (fine→coarse) would risk injecting local noise into the global representation before sufficient context has been established; bidirectional schemes could be explored in future work. Only adjacent scales exchange messages, as non-adjacent scales differ too greatly in temporal resolution for a single linear projection to provide meaningful alignment.

For each adjacent pair (s,s−1) where s−1 is finer:


$$\begin{array}{c} {𝐦}^{s\to s-1}={Pool}_{N_{s-1}}\;\left({𝐙}_{SAFCA}^{s}\right){𝐖}_{m}^{s}\in {\mathbb{R}}^{N_{s-1}\times d}, \end{array}$$ (17)

$$\begin{array}{c} {𝐙}_{ISIE}^{s-1}=LayerNorm\;\left(FFN\;\left({𝐙}_{SAFCA}^{s-1}+{𝐦}^{s\to s-1}\right)+{𝐙}_{SAFCA}^{s-1}\right). \end{array}$$ (18)

The coarsest stream is passed through unchanged. Fig 3 shows the schematic illustration of the proposed module.

**Fig 3:**
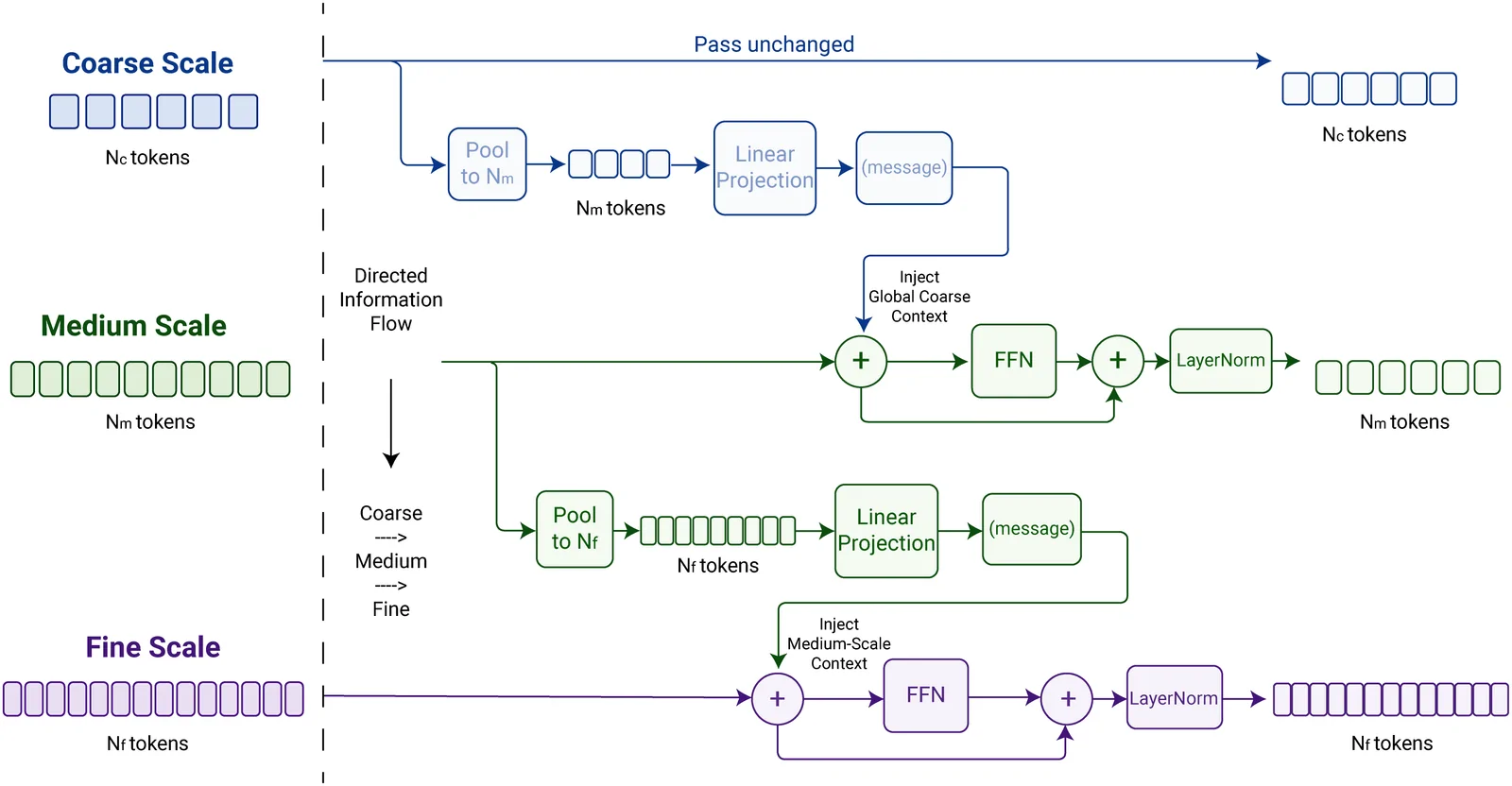
Schematic illustration of the proposed Inter-Scale Information Exchange (ISIE) module with coarse-to-fine message passing.

#### 3.4.3 Factorized temporal–channel attention (FTCA).

After ISIE, each scale’s token sequence ${𝐙}_{ISIE}^{s}\in {\mathbb{R}}^{N_{s}\times d}$ must model temporal dependencies across the $N_{s}$ token positions and feature-channel dependencies across the *d* embedding dimensions. FTCA factorizes these into two sequential operators to avoid the $O(N_{s}^{2}d)$ cost of joint attention.

**Temporal Attention.** A linear-attention operator is applied along the token dimension using the feature-map approximation ϕ(𝐱)=elu(𝐱)+1:


$$\begin{array}{c} LinAttn(𝐐,𝐊,𝐕{)}_{i}=\frac{\phi ({𝐪}_{i}{)}^{⊤}\;\left(\sum_{j}\phi ({𝐤}_{j}){𝐯}_{j}^{⊤}\right)}{\phi ({𝐪}_{i}{)}^{⊤}\sum_{j}\phi ({𝐤}_{j})}, \end{array}$$ (19)

where ${𝐪}_{i},{𝐤}_{j},{𝐯}_{j}\in {\mathbb{R}}^{d_{h}}$ and the cumulative sum $\sum_{j}\phi ({𝐤}_{j}){𝐯}_{j}^{⊤}$ can be maintained in $O(d_{h}^{2})$ memory for causal or $O(N_{s}d_{h}^{2})$ for full attention, reducing the $N_{s}$-dependent cost from $O(N_{s}^{2}d)$ to $O(N_{s}d)$:


$$\begin{array}{c} {𝐙}_{T}^{s}=LayerNorm\;\left({LinAttn}_{T}\;\left({𝐙}_{ISIE}^{s}\right)+{𝐙}_{ISIE}^{s}\right). \end{array}$$ (20)

**Channel Attention.** To capture dependencies across the *d* embedding dimensions, we reshape ${𝐙}_{T}^{s}$ so that features become the sequence dimension. Concretely, we transpose to treat each of the *d* feature channels as a “token” of length $N_{s}$:


$$\begin{array}{c} {\hat{𝐙}}^{s}={\left({𝐙}_{T}^{s}\right)}^{⊤}\in {\mathbb{R}}^{d\times N_{s}}, \end{array}$$ (21)

apply multi-head self-attention with *H* heads of size $N_{s}/H$ along the *d* token dimension:


$$\begin{array}{c} {\bar{𝐙}}^{s}={MHA}_{d}\;\left({\hat{𝐙}}^{s}\right)\in {\mathbb{R}}^{d\times N_{s}}, \end{array}$$ (22)

then transpose back:


$$\begin{array}{c} {𝐙}_{C}^{s}=LayerNorm\;\left({\left({\bar{𝐙}}^{s}\right)}^{⊤}+{𝐙}_{T}^{s}\right)\in {\mathbb{R}}^{N_{s}\times d}. \end{array}$$ (23)

**FFN Refinement.**


$$\begin{array}{c} {𝐙}_{out}^{s}=LayerNorm\;\left(FFN\;\left({𝐙}_{C}^{s}\right)+{𝐙}_{C}^{s}\right). \end{array}$$ (24)

Fig 4 shows the schematic illustration of the proposed Factorized Temporal–Channel Attention module.

**Fig 4:**
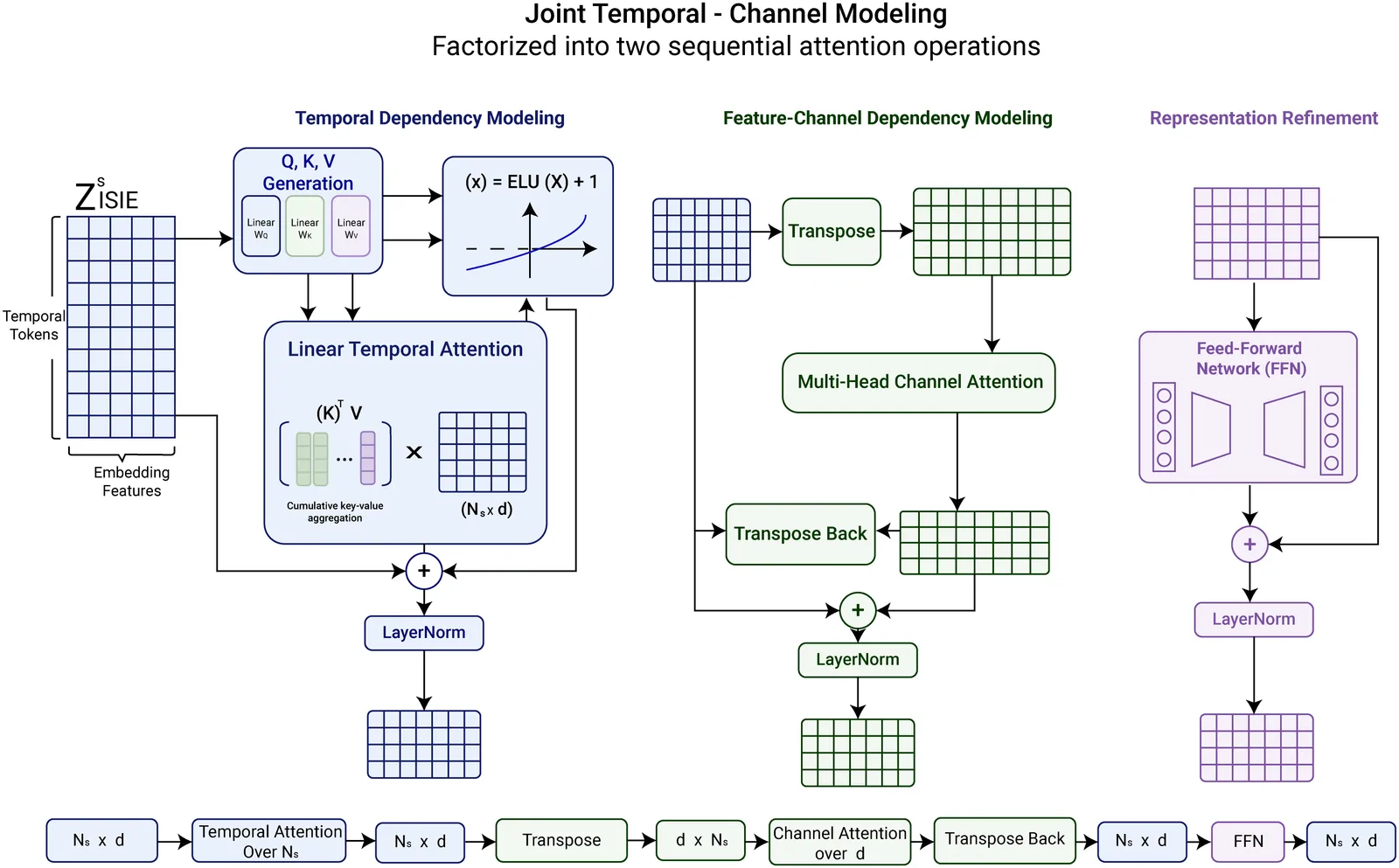
Schematic illustration of the proposed Factorized Temporal–Channel Attention (FTCA) module.

### 3.5 Multi-scale aggregation and classification

**Intra-Scale Attention Pooling.** Each token sequence is collapsed to a fixed vector:


$$\begin{array}{c} \alpha^{s}=softmax\;\left(\frac{{𝐙}_{out}^{s}\;{𝐰}_{\alpha }}{\sqrt{d}}\right)\in {\mathbb{R}}^{N_{s}},\;{𝐳}^{s}={\left({𝐙}_{out}^{s}\right)}^{⊤}\alpha^{s}\in {\mathbb{R}}^{d}. \end{array}$$ (25)

**Inter-Scale Fusion.** Learnable scale weights are computed from the concatenated pooled vectors:


$$\begin{array}{c} \beta =softmax\;\left({𝐖}_{\beta }\;Concat[{𝐳}^{f},{𝐳}^{m},{𝐳}^{c}]\right)\in {\mathbb{R}}^{3},\;{𝐖}_{\beta }\in {\mathbb{R}}^{3\times 3d}, \end{array}$$ (26)

$$\begin{array}{c} 𝐳=\beta_{f}\;{𝐳}^{f}+\beta_{m}\;{𝐳}^{m}+\beta_{c}\;{𝐳}^{c}\in {\mathbb{R}}^{d}. \end{array}$$ (27)

**Classification Head.** A 3-layer MLP produces activity logits:


$$\begin{array}{c} \hat{𝐲}={𝐖}_{3}\;GELU\;\left({𝐖}_{2}\;GELU\;\left({𝐖}_{1}\;𝐳\right)\right)\in {\mathbb{R}}^{K}. \end{array}$$ (28)

Softmax is applied for multi-class tasks; sigmoid for binary.

### 3.6 Training strategy

**Primary Loss.** Label-smoothed cross-entropy with $\ell_{2}$ regularization:


$$\begin{array}{c} {ℒ}_{CE}=-\sum_{k=1}^{K}{\tilde{y}}_{k}\log{\hat{y}}_{k},\;{\tilde{y}}_{k}=(1-\epsilon )\;y_{k}+\frac{\epsilon }{K},\;\epsilon =0.1. \end{array}$$ (29)

Per-class frequency weights $w_{k}\propto 1/n_{k}$ are applied on imbalanced datasets.

**Scale-Utilization Regularizer.** A key design assumption is that the three scales contribute complementarily to the final prediction. If the learned scale weights β concentrate on a single scale, the multi-scale design degenerates. We prevent this with an entropy-maximizing regularizer over β:


$$\begin{array}{c} {ℒ}_{SU}=-H(\beta )=\sum_{s\in \{f,m,c\}}\beta_{s}\log\beta_{s}. \end{array}$$ (30)

Minimizing ${ℒ}_{SU}$ (i.e., maximizing entropy) encourages balanced utilization across scales rather than enforcing strict uniformity. Some datasets may genuinely benefit from one scale more than others; the task loss ${ℒ}_{CE}$ retains that flexibility by driving each scale’s representation to be discriminative, so the two objectives together encourage *all three scales to be individually informative* rather than collapsed onto a single dominant scale.

**Total Objective.**


$$\begin{array}{c} ℒ={ℒ}_{CE}+\lambda_{1}\;{ℒ}_{SU}+\lambda_{2}\;\|\theta {\|}_{2}^{2}, \end{array}$$ (31)

with $\lambda_{1}=0.1$ and $\lambda_{2}=10^{-4}$.

**Optimization.** Batch size (64) and 100 epochs with AdamW is used ($\beta_{1}=0.9$, $\beta_{2}=0.999$, lr 10^–3^) with cosine annealing and warm restarts. In transformer blocks Dropout is applied 0.1 and in the classifier it is applied 0.2.

### 3.7 Model complexity analysis

**Complexity Derivations.** Per encoder block and per scale the asymptotic costs are derived as Let $N=\max(N_{f},N_{m},N_{c})$ denote the maximum token count.

- **UCH:** Per-channel descriptor extraction (Eq. 2) computes spectral power bins via FFT, costing 𝒪(TlogT) per channel and 𝒪(CTlogT) across all *C* channels. The shared MLP scorer (Eq. 3) costs $𝒪(Cd_{\psi })$, independent of *T*. The soft-weighted group projection and Conv1D projection (Eqs. 4–5) map each of the *M* groups from *C* to *d*/*M* channels at every timestep, costing 𝒪(TCd) in total. The fusion step (Eq. 6) applies a d×d linear projection at each timestep, costing $𝒪(Td^{2})$. Since d≥C across all benchmarks used here (C≤113≤d=128), the 𝒪(TCd) and $𝒪(Td^{2})$ terms are comparable in order to AMSPE’s $𝒪(Td^{2})$ cost below, rather than negligible.
- **AMSPE:** this module employs Conv1D with kernel $p_{s}$ and stride ${str}_{s}=p_{s}/2$ with input T×d and output $N_{s}\times d$ where $N_{s}\approx 2T/p_{s}$. Cost $=N_{s}\cdot p_{s}\cdot d\cdot d=(2T/p_{s})\cdot p_{s}\cdot d^{2}=O(Td^{2})$, to embedding dimension *d*. This assumes a dense projection and because *d* is fixed across all the datasets, this reduce to *O*(*T*).
- **SAFCA Fourier:** As this module applies a Fourier-based mixing instead of standard self-attention so it apply 1D FFT over $N_{s}$ tokens, and *d* channels processing and $O(N_{s}\log N_{s}\cdot d)$. Spectral filter application: $O(N_{s}d)$ to filter them together, the total computational cost becomes O(NdlogN).
- **ISIE:** This module has mainly two operations Pooling *O*(*N d*) and linear projection *O*(*N d*^2^). So the total cost, which is linear and cheap, is *O*(*N d*^2^).
- **FTCA Temporal:** This module avoid computing the full matrix so the linear attention costs *O*(*N d*) (the cumulative sum is *O*(*d*^2^) memory and *O*(*N d*^2^) estimate, but with N≫d controlled by *O*(*N d*) per head). the total complexity is *O*(*N d*).
- **FTCA Channel:** This module selects across channels instead of across time, so it transposes to $d\times N_{s}$, Multi-head attention on *d* tokens of size $N_{s}/H$: $O(d^{2}\cdot N_{s}/H\cdot H)=O(d^{2}N_{s})$. so the final complexity is *O*(*d*^2^ *N*).

The above derivations are summed up in Table 1.

**Table 1:** Asymptotic computational complexity per encoder block per scale. *N*: max token count; *d*: embedding dim; *T*: time-steps; *C*: channel count; $p_{s}$: patch size.

Module	Dominant operation	Cost
UCH	Feature encoding	$O(CT\log T+TCd+Td^{2})$
AMSPE	Conv1D patching	*O*(*Td*^2^)
SAFCA (Fourier mix)	FFT + spectral filter	O(NdlogN)
SAFCA (Cross-attn)	Dot-product attention	*O*(*N*^2^ *d*)
ISIE	Linear proj. + pooling	*O*(*Nd*^2^)
FTCA (Temporal)	Linear attention	*O*(*Nd*)
FTCA (Channel)	MHA over *d* dimension	*O*(*d*^2^ *N*)

The complete forward pass is explained in Algorithm 1.

**Algorithm 1 UniHAR Forward Pass**

**Require:** Sensor window $𝐗\in {\mathbb{R}}^{T\times C}$, sampling rate $f_{s}$

**Ensure:** Activity logits $\hat{𝐲}\in {\mathbb{R}}^{K}$

1:  **// Stage 1: Universal Channel Harmonization**

2:  **for** *c* = 1 to *C* **do**

3:   $\psi_{c}\leftarrow [\mu_{c},\sigma_{c},\gamma_{1,c},\gamma_{2,c},{𝐩}_{c}^{⊤}{]}^{⊤}$               *(*Eq. 2*)*

4:   ${𝐚}_{c}\leftarrow softmax({MLP}_{g}(\psi_{c}))$                *(*Eq. 3*)*

5:  **end for**

6:  $𝐀\leftarrow [{𝐚}_{1},...,{𝐚}_{C}{]}^{⊤}$

7:  **for** *m* = 1 to *M* **do**

8:   ${𝐇}_{m}\leftarrow Conv1D(𝐗\;diag({𝐀}_{:,m}),\;d/M)$*            (*Eqs. 4*–*5*)*

9:  **end for**

10:  ${𝐗}_{u}\leftarrow LayerNorm({𝐖}_{f}\;Concat[{𝐇}_{1},...,{𝐇}_{M}])$           *(*Eq. 6*)*

11:  **// Stage 2: Adaptive Multi-Scale Patch Embedding**

12:  $\left\{p_{s},{str}_{s}{\}}_{s}\leftarrow ComputeScales(T,f_{s}\right)$               *(*Eq. 7*)*

13:  **for** s∈{f,m,c} **do**

14:   ${𝐙}^{s}\leftarrow Conv1D({𝐗}_{u};p_{s},{str}_{s})+{PE}^{s}+{SE}^{s}+TE$         *(*Eqs. 8*–*9*)*

15:  **end for**

16:  **// Stage 3: Hierarchical Transformer Encoder**

17:  **for** ℓ=1 to *L* **do**

18:   $\left\{{𝐙}^{s}\}\leftarrow SAFCA({𝐙}^{f},{𝐙}^{m},{𝐙}^{c}\right)$                *(*Eqs. 10*–*16*)*

19:   $\left\{{𝐙}^{s}\}\leftarrow ISIE({𝐙}^{f},{𝐙}^{m},{𝐙}^{c}\right)$                 *(*Eqs. 17*–*18*)*

20:   $\left\{{𝐙}^{s}\}\leftarrow FTCA({𝐙}^{f},{𝐙}^{m},{𝐙}^{c}\right)$                *(*Eqs. 20*–*24*)*

21:  **end for**

22:  **// Stage 4: Aggregation and Classification**

23:  **for** s∈{f,m,c} **do**

24:   ${𝐳}^{s}\leftarrow AttnPool({𝐙}^{s})$*                   (*Eq. 25*)*

25:  **end for**

26:  $𝐳\leftarrow ScaleFuse({𝐳}^{f},{𝐳}^{m},{𝐳}^{c})$                 *(*Eqs. 26*–*27*)*

27:  $\hat{𝐲}\leftarrow MLP(𝐳)$                       *(*Eq. 28*)*

28:  **return** $\hat{𝐲}$

### 3.8 Experimental configuration

Before presenting the experimental results, we summarize the dataset-dependent input configuration and the shared UniHAR architecture and training hyperparameters. UniHAR uses the same backbone configuration across all four datasets. Only the input window length, sampling rate, number of input channels, and number of output classes vary according to the dataset. The Adaptive Multi-Scale Patch Embedding (AMSPE) patch sizes are calculated from the dataset sampling rate using the target durations $\tau_{f}=0.15$ s, $\tau_{m}=0.35$ s, and $\tau_{c}=0.70$ s. Table 2 presents the dataset-dependent settings, while Table 3 summarizes the shared network and optimization settings.

**Table 2:** Dataset-dependent input and AMSPE configurations. Here, $f_{s}$ denotes the sampling rate, *T* is the number of samples in an input window, *C* is the post-preprocessing input-channel count, *K* is the number of activity classes, and $N_{f}$, $N_{m}$, and $N_{c}$ are the numbers of fine-, medium-, and coarse-scale tokens, respectively. The target patch durations are fixed to $\left(\tau_{f},\tau_{m},\tau_{c})=(0.15,0.35,0.70\right)$ s for all datasets.

Dataset	$f_{s}$ (Hz)	T	C	K	Window (s)	$p_{f}/p_{m}/p_{c}$	$N_{f}/N_{m}/N_{c}$	Overlap
UCI-HAR	50	128	9	6	2.56	7/17/35	41/14/6	50%
Opportunity	30	90	113	18	3.00	4/10/21	44/17/7	50%
Daphnet	64	192	9	2	3.00	9/22/44	46/16/7	50%
PAMAP2	100	256	18	12	2.56	15/35/70	35/14/6	50%

**Table 3:** Shared UniHAR architecture and training configuration used across the evaluated datasets.

Component	Hyperparameter	Setting
Input/UCH	Maximum supported channel count $C_{\max}$	113
	Per-channel descriptor dimension $d_{\psi }$	16
	UCH group count *M*	4
	Group-assignment MLP	16→32→4
	Unified embedding dimension *d*_model_	128
	Per-group projection dimension *d*_model_/*M*	32
AMSPE	Number of temporal scales	3
	Target patch durations	0.15/0.35/0.70 s
	Patch stride	$\max\left(\left\lfloor p_{s}/2\right\rfloor ,2\right)$
	Patch-embedding output dimension	128
Hierarchical encoder	Number of encoder blocks *L*	4
	Number of attention heads $n_{h}$	8
	Per-head dimension $d_{h}$	16
	Feed-forward dimension *d*_FFN_	512
	Feed-forward expansion ratio	4×
	Transformer dropout	0.1
	Normalization	Layer normalization
	Residual connections	Applied after each sub-layer
SAFCA	Fourier-transform dimension	Temporal-token dimension
	Number of temporal scales exchanged	3
	Cross-scale fusion	Learnable gated fusion
ISIE	Message-passing direction	Coarse → medium → fine
	Scale connections	Adjacent scales only
FTCA	Temporal-attention mechanism	Linear attention with ϕ(x)=ELU(x)+1
	Number of channel-attention heads	8
	Attention implementation dimension	128
	Per-head attention dimension	16
Classifier	Number of fully connected layers	3
	Hidden-layer dimensions	128→256→128→K
	Activation function	GELU
	Classifier dropout	0.2
Optimization	Optimizer	AdamW
	Initial learning rate	10^–3^
	Adam coefficients	$\beta_{1}=0.9$, $\beta_{2}=0.999$
	Learning-rate scheduler	Cosine annealing with warm restarts
	Batch size	64
	Maximum number of training epochs	100
	Early-stopping patience	15 epochs
	Label-smoothing coefficient	0.1
	Number of independent runs	5 random seeds
	Class weighting	Inverse-frequency weighting for imbalanced datasets

The patch sizes in Table 2 are obtained as


$$\begin{array}{cc} p_{f} & =\max\left(4,\left\lfloor 0.15f_{s}\right\rfloor \right),\\ p_{m} & =\max\left(8,\left\lfloor 0.35f_{s}\right\rfloor \right),\\ p_{c} & =\max\left(16,\left\lfloor 0.70f_{s}\right\rfloor \right). \end{array}$$ (32)

The scale-specific stride is defined as


$$\begin{array}{c} {\mathrm{str}}_{s}=\max\left(\left\lfloor \frac{p_{s}}{2}\right\rfloor ,2\right). \end{array}$$ (33)

The resulting number of tokens at scale *s* is


$$\begin{array}{c} N_{s}=\left\lfloor \frac{T-p_{s}}{{\mathrm{str}}_{s}}\right\rfloor +1. \end{array}$$ (34)

All architecture-related settings, including *d*_model_, *M*, *L*, $n_{h}$, and *d*_FFN_, are held constant across datasets. The UCH group-assignment scorer uses a two-layer architecture of 16→32→4, where the four output values represent the soft channel-group assignments. The unified feature dimension is *d*_model_ = 128, and each of the four UCH groups produces a 32-dimensional feature representation.

The hierarchical encoder contains four identical blocks and uses eight attention heads, resulting in a per-head dimension of $d_{h}=d_{model}/n_{h}=16$. The feed-forward network expands the 128-dimensional representation to 512 dimensions before projecting it back to 128 dimensions. The classification head subsequently maps the aggregated representation through dimensions 128→256→128→K.

Dataset-specific output layers are used only to accommodate the corresponding number of activity classes *K*. No changes are made to the UCH, AMSPE, SAFCA, ISIE, or FTCA backbone when moving between datasets.

### 3.9 Data preprocessing

A standardized pre-treatment was applied to the data from the UCI-HAR, Opportunity, Daphne, and PAMAP2, which included a process of channel selection, the elimination of samples with invalid or incorrect values, and the conversion of data from one channel to another, followed by windowed blocks. All these data were kept at their original sampling rate because AMSPE determines the size of the patches from the physical sampling frequency. To avoid the loss of information, independent data samples were generated before the calculation of pre-treatment statistics, and the normalization parameters were only estimated from the training set.

For UCI-HAR [54], nine accelerometer and gyroscope channels were retained. The 50-Hz signals were represented using 2.56-s windows of 128 samples with 50% overlap. The original six activity classes and official subject-independent split were preserved.

For Opportunity [55], timestamps, annotation fields, and non-sensor metadata were removed, while 113 sensor channels and 18 activity classes were retained. The 30-Hz signals were segmented into 3-s windows of 90 samples with a stride of 45 samples.

For Daphnet [56], nine accelerometer channels from the ankle, thigh, and trunk were used. The 64-Hz recordings were divided into 3-s windows of 192 samples with 50% overlap. The two activity labels were mapped to contiguous class indices.

For PAMAP2 [57], tri-axial accelerometer and gyroscope signals from the hand, chest, and ankle were selected, producing 18 channels. Samples with undefined activity identifier zero were removed. The 100-Hz signals were segmented into 2.56-s windows of 256 samples with a stride of 128 samples.

PAMAP2 missing values were processed independently for each subject, recording, and sensor channel before window segmentation. Invalid and infinite values were first converted to NaN. Internal gaps were filled using linear interpolation:


$$\begin{array}{c} {\tilde{x}}_{t,c}=x_{t_{a},c}+\frac{t-t_{a}}{t_{b}-t_{a}}\left(x_{t_{b},c}-x_{t_{a},c}\right), \end{array}$$ (35)

where $t_{a}$ and $t_{b}$ are the nearest valid samples before and after the missing interval. Leading and trailing missing values were replaced by the nearest valid observation using backward and forward filling, respectively. Windows containing remaining non-finite values or channels that were entirely unavailable were discarded.

Finally, each channel was standardized using training-set statistics:


$$\begin{array}{c} z_{t,c}=\frac{x_{t,c}-\mu_{c}^{train}}{\sigma_{c}^{train}+\varepsilon },\;\varepsilon =10^{-8}. \end{array}$$ (36)

The same statistics were applied to the validation and test sets. For sample-level annotations, each window received its majority activity label, with the center-sample label used to resolve ties. Windows were constructed independently within each subject and recording and retained their dataset-specific dimensions T×C for subsequent processing by UCH.

### 3.10 Data partitioning and model selection

A subject-independent partitioning was used to measure generalization to unseen participants. All the windows corresponding to a given subject were assigned to either the training, validation or test set, so that no subject appeared in more than one part. The partitioning was carried out before normalization and the generation of the individual windows to prevent any leakage of information. The same partitioning was used for all unrelated subjects and ablation experiments. For UCI-HAR, the official partitioning was used: 21 subjects formed the development set, and the nine official test subjects the test set. Four subjects in the development set were set aside for testing, yielding 17 training, 4 testing, and 9 test subjects. For OPP, subjects S1 and S2 were used for training, S3 for validation, and S4 only for testing. For Daphnet, the ten participants were divided into seven training, one validation and two test subjects. For PAMAP, six training, one testing, and two test subjects. The Daphnet and PAMAP subjects were assigned by drawing lots with a known random seed of 42. Thus, the test set consisted of valid windows from subjects kept separate for testing rather than windows randomly selected from those seen during training.

The checkpoint with the lowest validation loss was selected. When validation losses were equal, the checkpoint with the higher validation macro-F1 was selected. When two checkpoints had the same averaged loss in the test set, the one with the highest macro-F1 score was chosen. The selected weights were only reinstated at the end of the training and were then run only once on the test set. The test set was not used for hyperparameter tuning, stopping criteria, checkpoint selection, or normalization of metrics.

## 4 Results

The comprehensive evalution is performed on four widely used HAR benchmarks: UCI-HAR [54], Opportunity [55], Daphnet Freezing of Gait [56], and PAMAP2 [57]. These datasets consist of smartphone- and IMU-based sensing of human activities, with coarse-grained, varying activity levels. These datasets correspond to challenging scenarios such as pathological motion and severe class imbalance. The subject-independent evaluation is performed on all datasets.

### 4.1 Baseline models and efficiency evaluation

To ensure a fair comparison, all competing models were reimplemented and evaluated under the same experimental conditions as UniHAR. These models included CNN [24], CNN–LSTM [25], vanilla LSTM (vLSTM), stacked LSTM (sLSTM), and bidirectional LSTM (BiLSTM) [26],Inception-GRU [58], iSPLInception [29], DKInception [59] and Rep-LOM [30]. Their architectures were reproduced according to the corresponding publications, while dataset-specific classification layers were adjusted only to match the number of activity classes.

All models used the same preprocessed sensor windows, subject-independent training, validation, and test partitions, channel normalization, class weights, batch size, optimizer settings, early-stopping criterion, and checkpoint-selection procedure. Each model was trained from scratch, and the checkpoint with the lowest validation loss was evaluated once on the held-out test set. Therefore, the results in Tables 2–Table 5 were obtained using identical data partitions and evaluation protocols rather than being copied from the original publications.

**Table 4:** Dataset-specific preprocessing configurations.

Dataset	$f_{s}$ (Hz)	Duration (s)	T	C	K
UCI-HAR	50	2.56	128	9	6
Opportunity	30	3.00	90	113	18
Daphnet	64	3.00	192	9	2
PAMAP2	100	2.56	256	18	12

**Table 5:** Subject-independent data partitioning used for each dataset.

Dataset	Training subjects	Validation subjects	Test subjects
UCI-HAR	17	4	9
Opportunity	2	1	1
Daphnet	7	1	2
PAMAP2	6	1	2

In addition to accuracy and macro-F1 score, computational efficiency was evaluated using the number of trainable parameters, multiply–accumulate operations (MACs), inference latency, and throughput. Parameters and MACs were calculated for one dataset-specific input window. Inference latency was measured with batch size one in evaluation mode after 100 warm-up iterations, followed by 1000 timed forward passes. Throughput was measured with batch size and reported as the number of sensor windows processed per second. Timing excluded data loading and preprocessing and was repeated ten times, with the average result reported. Tables 6 and Table 7 show the computational efficiency metrics.

**Table 6:** Computational efficiency on Daphnet and PAMAP2. Parameters are reported in millions and MACs per input window.

Method	Daphnet (192×9)				PAMAP2 (256×18)			
	Params	MACs	Latency	Throughput	Params	MACs	Latency	Throughput
	(M)	(M)	(ms)	(win/s)	(M)	(M)	(ms)	(win/s)
CNN [24]	0.629	21.5	0.94	1024	0.837	35.8	1.22	823
CNN–LSTM [25]	4.026	102.7	4.81	206	4.029	138.3	6.25	161
vLSTM [26]	0.084	16.2	2.02	489	0.099	24.5	2.83	351
sLSTM [26]	0.263	50.3	3.42	293	0.267	67.3	4.60	212
BiLSTM [26]	0.167	31.9	3.24	315	0.196	49.2	4.40	227
iSPLInception [29]	1.327	46.8	1.92	517	1.339	65.2	2.38	416
Inception–GRU [58]	1.606	62.4	2.24	446	1.648	82.7	2.86	350
Rep-LOM [30]	0.354	15.9	1.21	826	0.428	23.8	1.52	658
DKInception [59]	0.397	16.4	1.49	691	0.471	25.2	1.79	558
**UniHAR (Ours)**	**0.789**	**25.7**	**3.34**	**298**	**0.797**	**24.6**	**3.55**	**281**

**Table 7:** Computational efficiency on UCI-HAR and Opportunity. Parameters are reported in millions and MACs per input window. Latency and throughput represent batch-one inference on an NVIDIA A100.

Method	UCI-HAR (128×9)				Opportunity (90×113)			
	Params	MACs	Latency	Throughput	Params	MACs	Latency	Throughput
	(M)	(M)	(ms)	(win/s)	(M)	(M)	(ms)	(win/s)
CNN [24]	0.424	14.8	0.72	1389	0.311	18.6	0.82	1220
CNN–LSTM [25]	3.502	72.4	3.65	274	3.444	68.1	3.42	292
vLSTM [26]	0.084	10.8	1.42	704	0.120	10.8	1.31	763
sLSTM [26]	0.263	33.7	2.31	433	0.272	24.4	2.08	481
BiLSTM [26]	0.168	21.5	2.18	459	0.239	21.5	1.98	505
iSPLInception [29]	1.327	32.6	1.56	641	1.355	38.9	1.68	595
Inception–GRU [58]	1.612	41.7	1.88	532	1.684	47.9	2.03	493
Rep-LOM [30]	0.356	10.6	0.96	1042	0.521	15.2	1.08	926
DKInception [59]	0.398	11.8	1.13	885	0.611	17.6	1.28	781
**UniHAR (Ours)**	**0.782**	**22.9**	**2.74**	**365**	**0.805**	**27.8**	**3.12**	**321**

### 4.2 Per-dataset results

Our model achieves the highest F1-score and accuracy on every dataset; validation curves, confusion matrices, and ROC curves are shown in Figs 4–15 and Tables 7–11 report the results across all four benchmark datasets.

**Table 8:** Performance comparison on the UCI-HAR dataset.

Method	Acc. (%)	Loss	F1 (%)
CNN	93.66	0.2993	93
CNN-LSTM	92.44	0.3864	92
vLSTM	90.67	0.3344	91
sLSTM	91.62	0.4559	91
BiLSTM	93.51	0.2829	93
Inception-GRU	95.41	0.1627	95
Rep-LOM	95.97	0.1564	95
iSPLInception	95.09	0.1761	95
DKInception	95.70	0.1625	96
**UniHAR (Ours)**	**96.35**	**0.1520**	**96**

**Table 9:** Performance comparison on the Opportunity dataset.

Method	Acc. (%)	Loss	F1 (%)
CNN	78.72	0.8149	77
CNN-LSTM	81.00	0.5979	81
vLSTM	77.66	0.7905	78
sLSTM	81.70	0.6261	81
BiLSTM	79.83	0.6442	80
Inception-GRU	86.03	0.4989	86
Rep-LOM	88.74	0.5026	88
iSPLInception	88.14	0.4790	88
DKInception	87.48	0.5198	87
**UniHAR (Ours)**	**94.45**	**0.1850**	**94**

**Table 10:** Performance comparison on the Daphnet dataset.

Method	Acc. (%)	Loss	F1 (%)
CNN	91.89	0.2847	92
CNN-LSTM	93.11	0.2658	93
vLSTM	93.02	0.2479	93
sLSTM	90.24	0.2821	90
BiLSTM	92.19	0.2485	92
Inception-GRU	94.27	0.2231	94
Rep-LOM	93.33	0.2168	93
iSPLInception	93.52	0.2271	94
DKInception	94.00	0.2286	94
**UniHAR (Ours)**	**96.44**	**0.2080**	**96**

**Table 11:** Performance comparison on the PAMAP2 dataset.

Method	Acc. (%)	Loss	F1 (%)
CNN	86.13	0.6732	86
CNN-LSTM	87.29	0.7041	86
vLSTM	86.21	0.5938	87
sLSTM	86.12	0.6835	86
BiLSTM	86.84	0.5498	87
Inception-GRU	88.91	0.4711	88
Rep-LOM	91.74	0.4227	91
iSPLInception	89.09	0.4322	89
DKInception	89.72	0.4872	90
**UniHAR (Ours)**	**99.23**	**0.1117**	**99**

**Fig 5:**
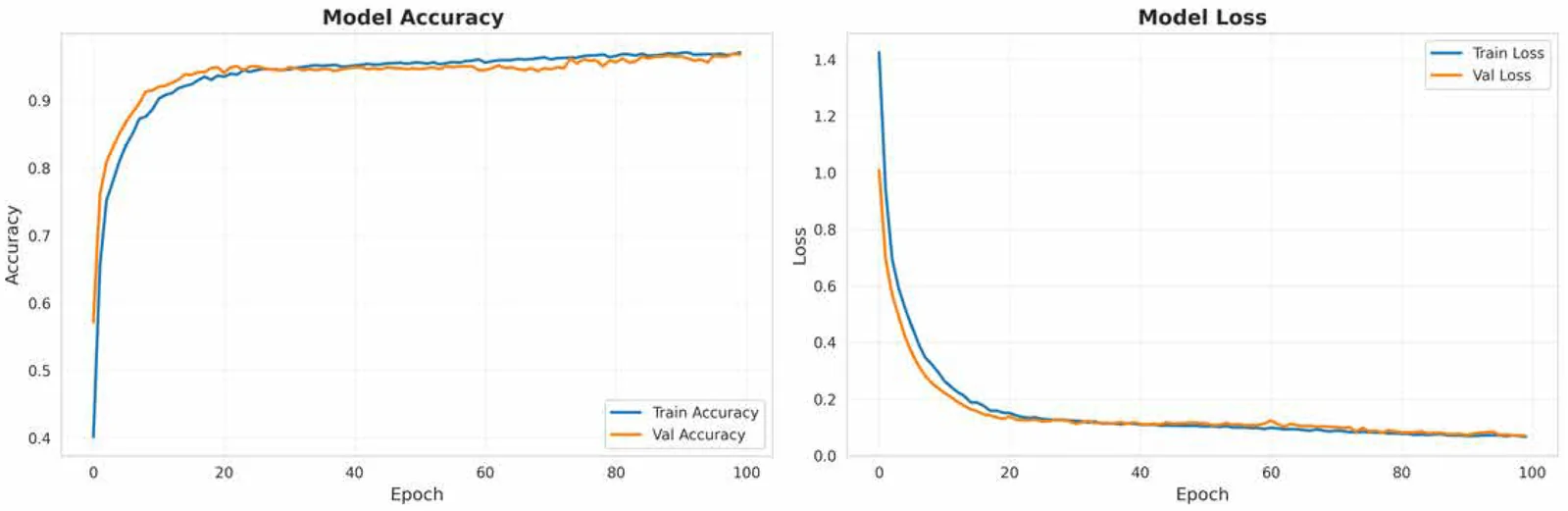
UniHAR results on the UCI-HAR dataset: training and validation curves illustrating the convergence behavior of the proposed UniHAR model.

**Fig 6:**
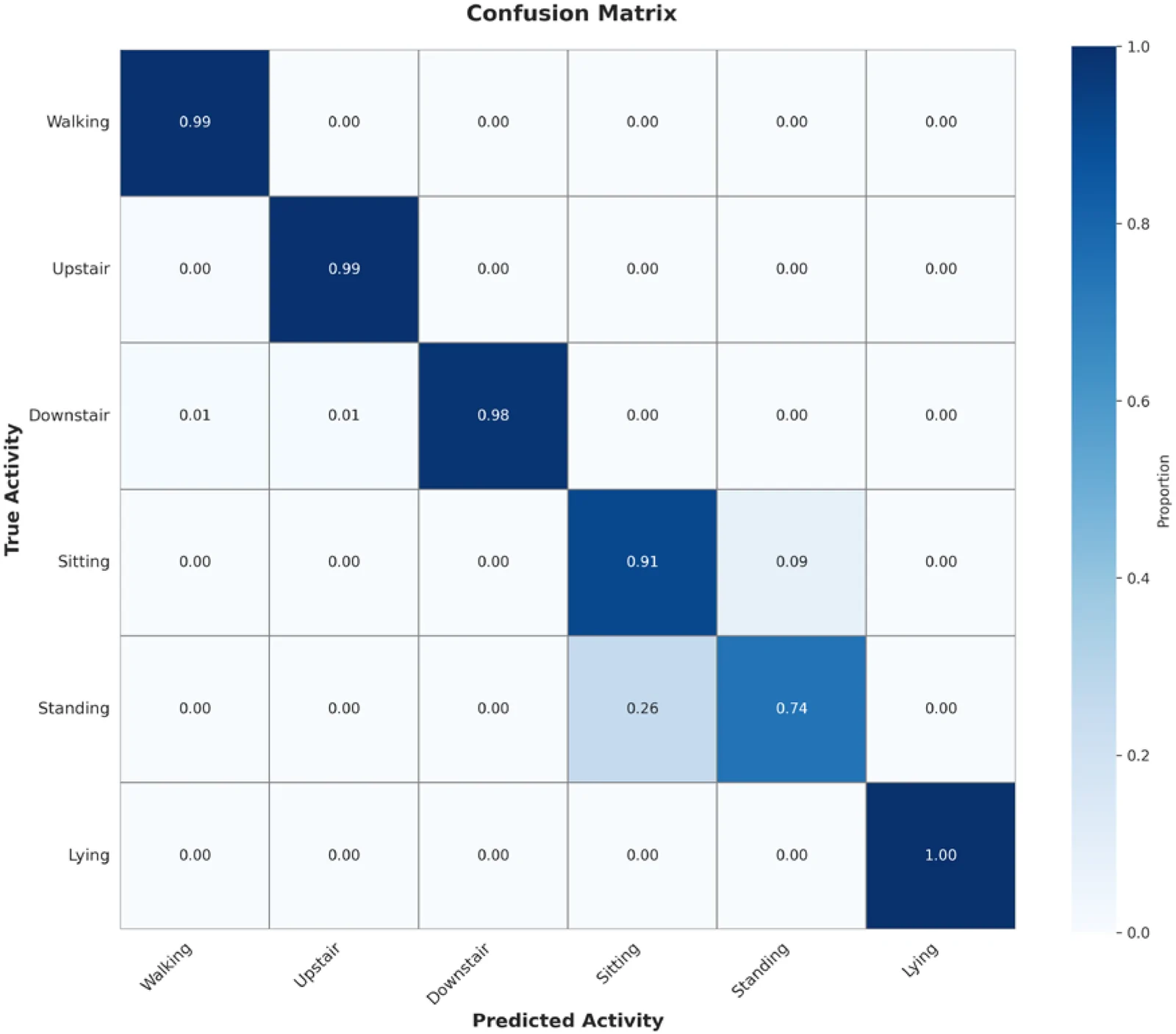
UniHAR results on the UCI-HAR dataset: confusion matrix shows the class-wise classification performance of the proposed model.

**Fig 7:**
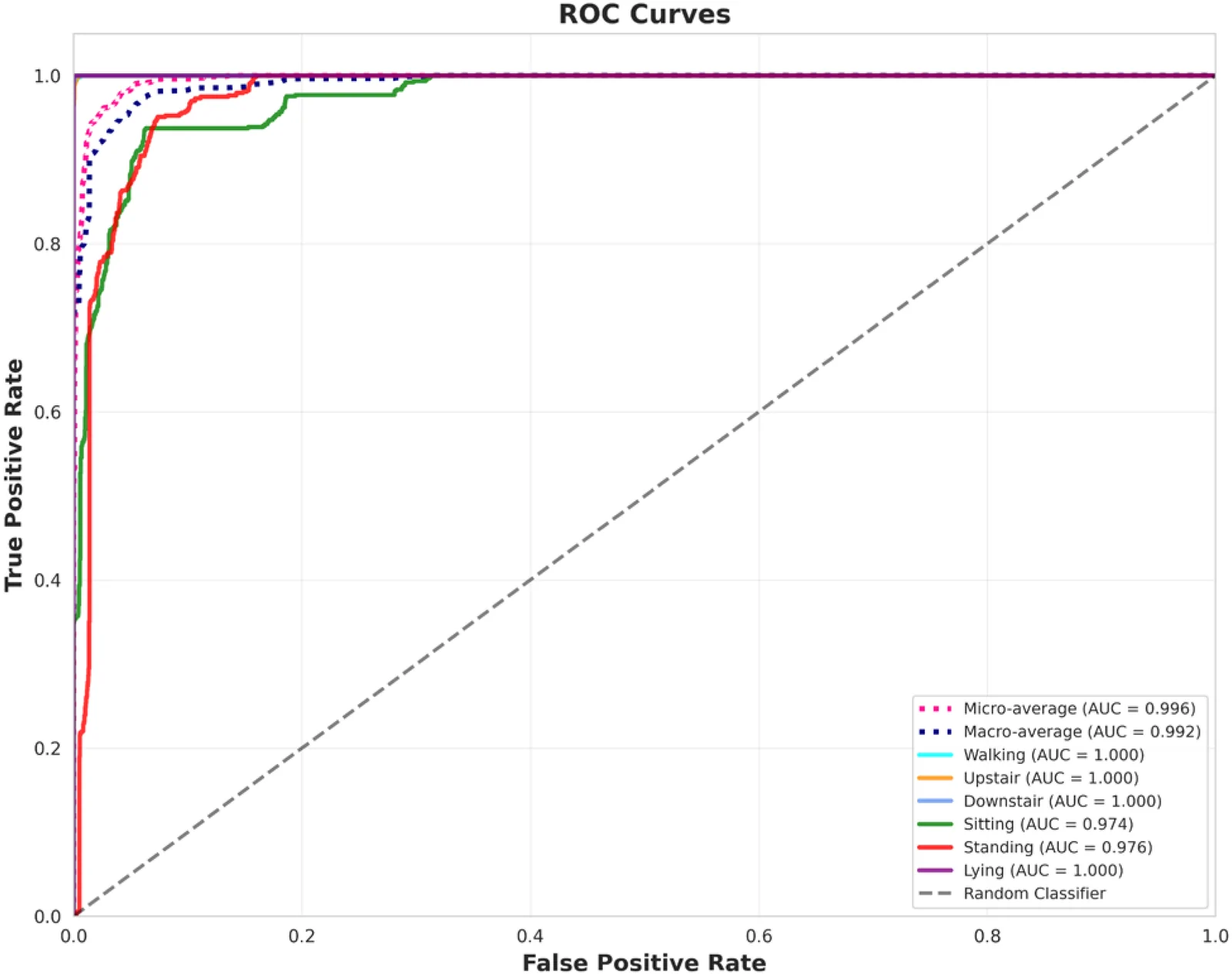
UniHAR results on the UCI-HAR dataset: (ROC) curves show the class-wise discrimination performance of the proposed model.

**Fig 8:**
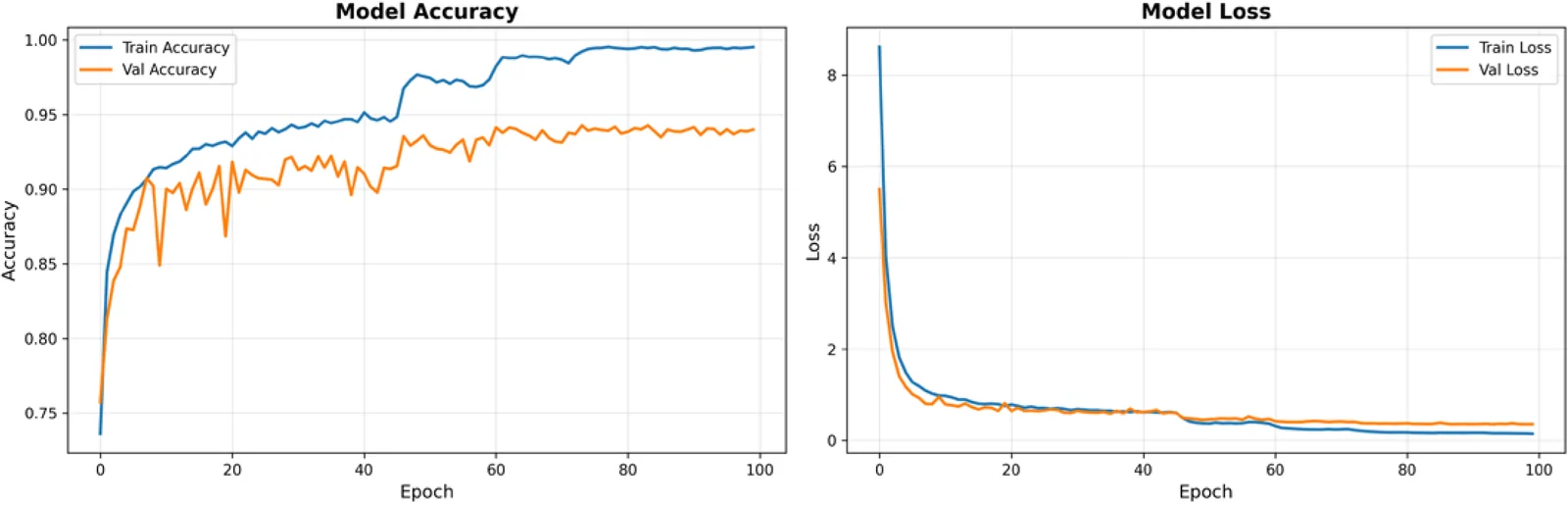
UniHAR results on the Opportunity dataset: training and validation curves illustrating the convergence behavior of the proposed UniHAR model.

**Fig 9:**
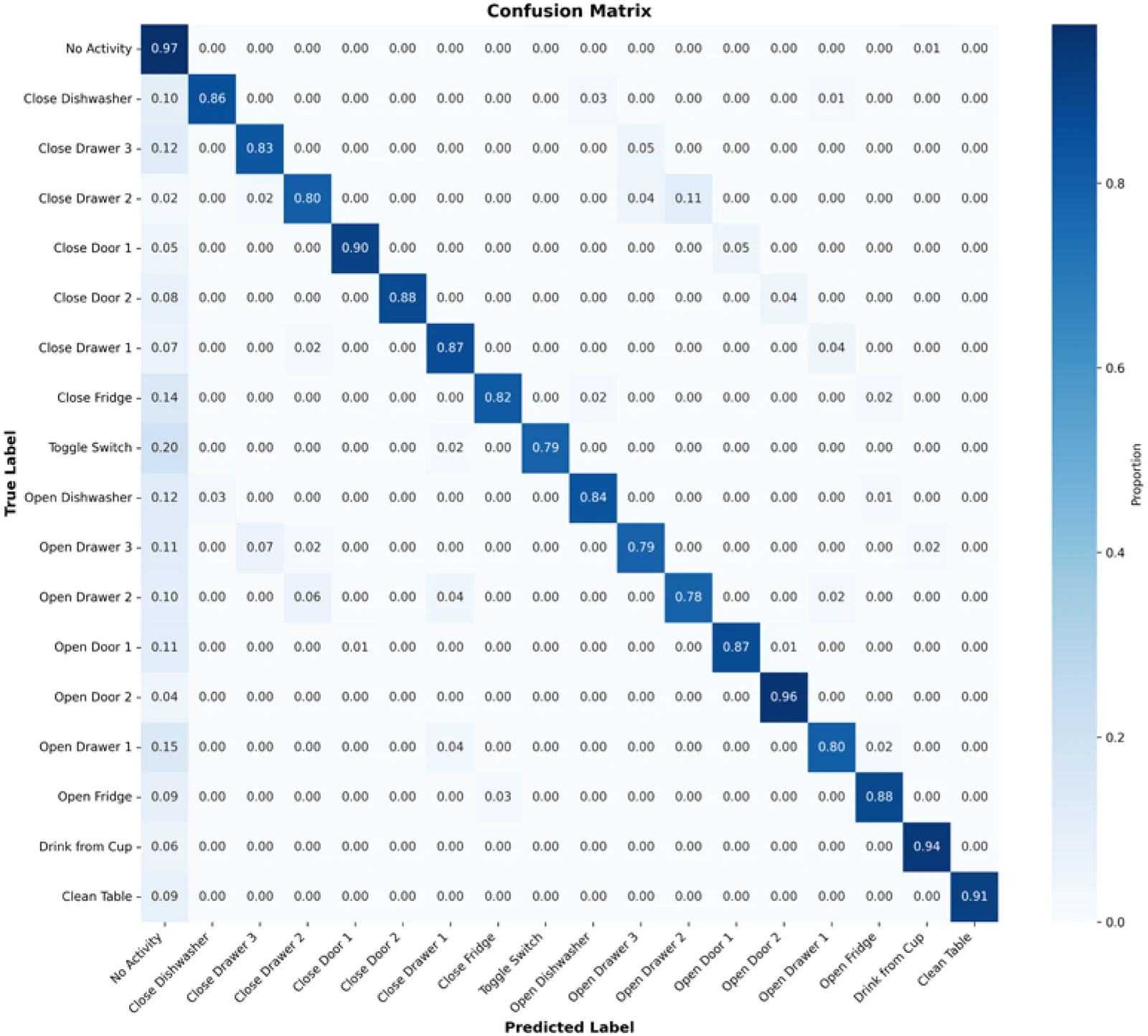
UniHAR results on the Opportunity dataset: confusion matrix shows the class-wise classification performance of the proposed model.

**Fig 10:**
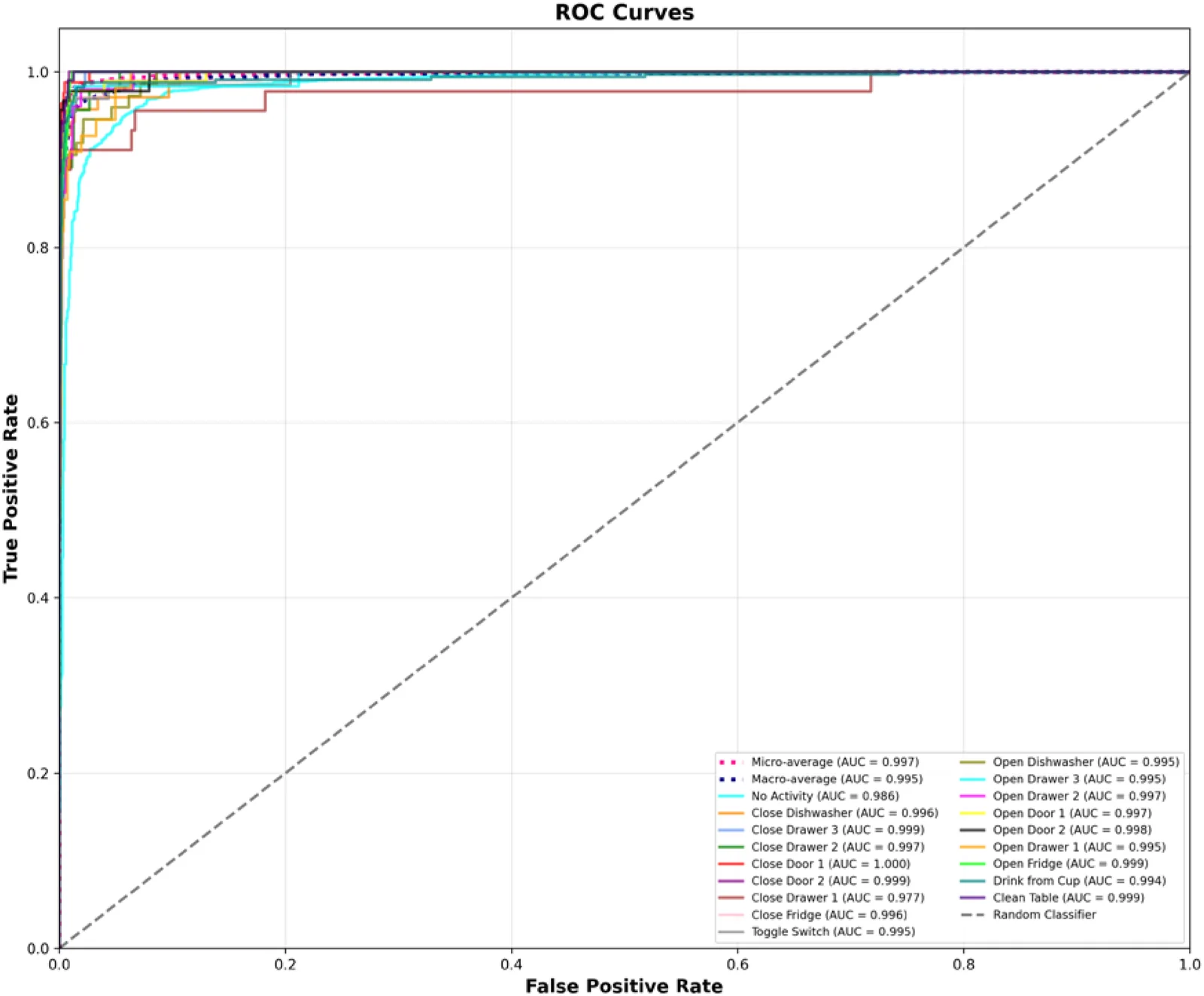
UniHAR results on the Opportunity dataset: (ROC) curves show the class-wise discrimination performance of the proposed model.

**Fig 11:**
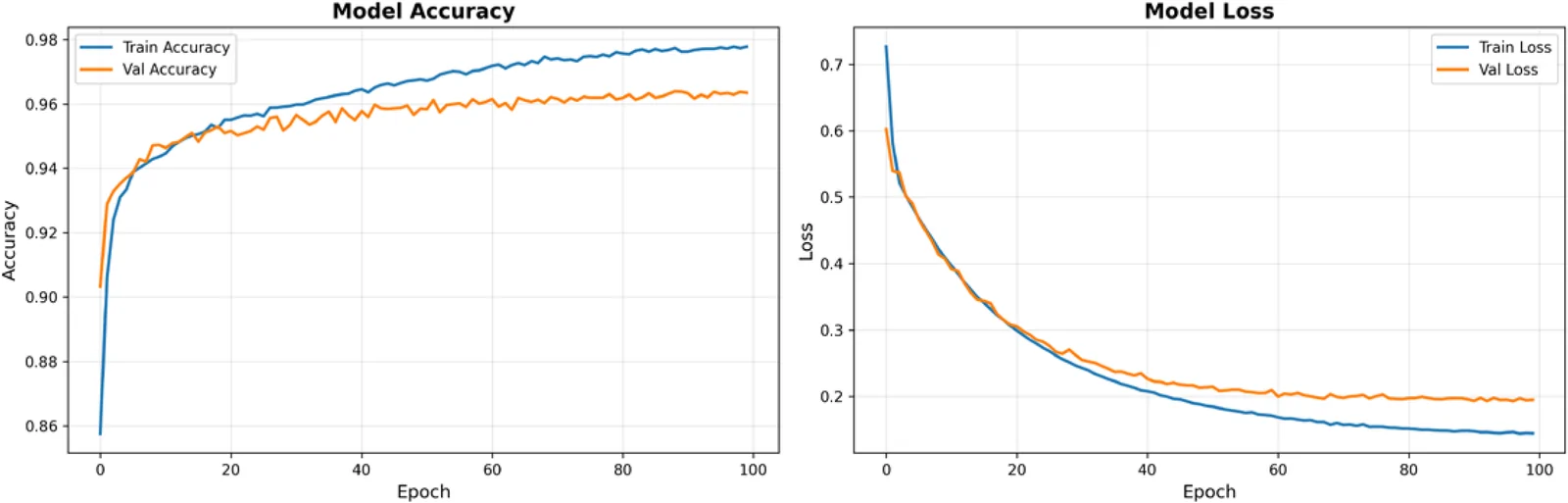
UniHAR results on the Daphnet dataset training and validation curves illustrating the convergence behavior of the proposed UniHAR model.

**Fig 12:**
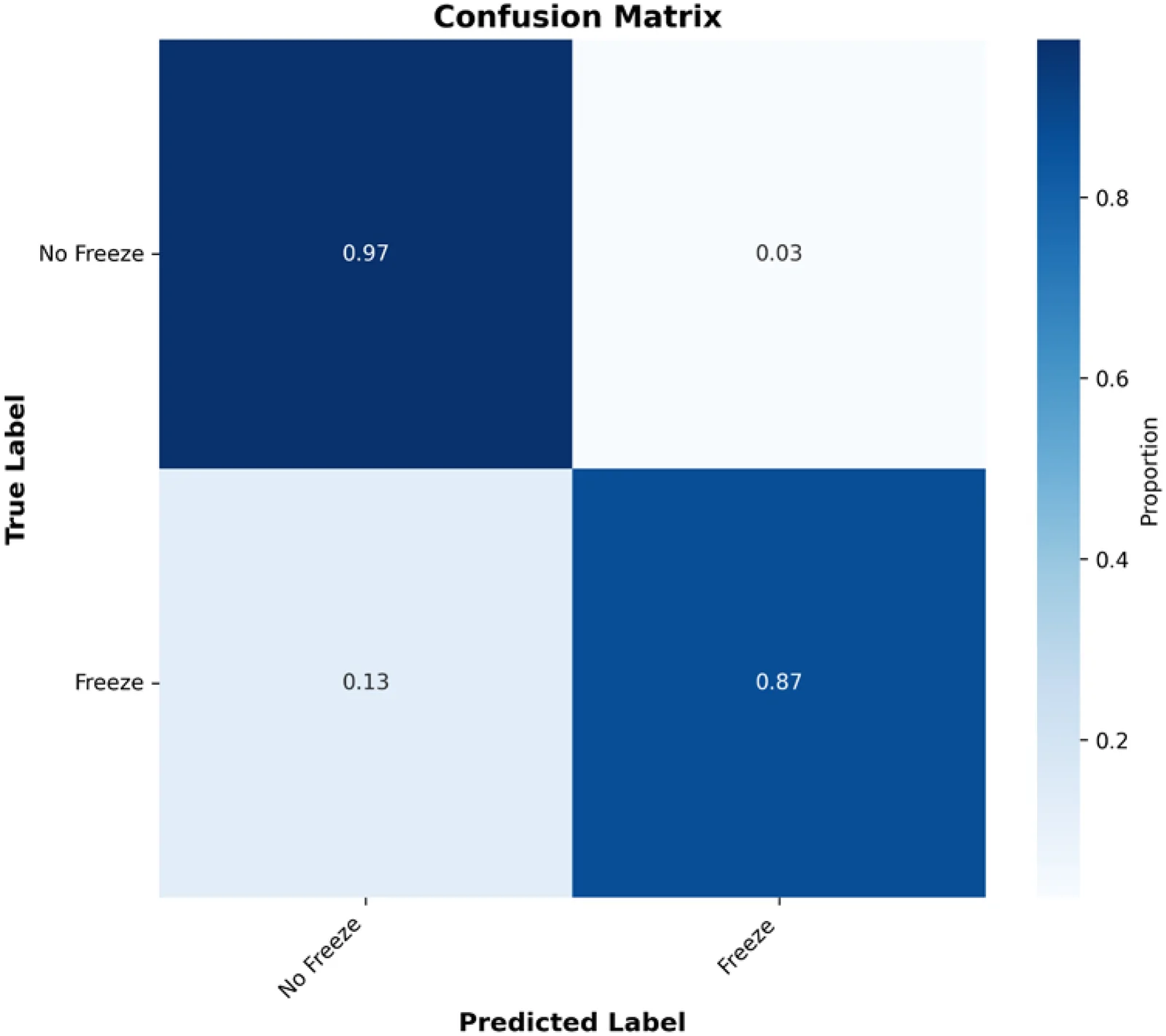
UniHAR results on the Daphnet dataset: confusion matrix shows the class-wise classification performance of the proposed model.

**Fig 13:**
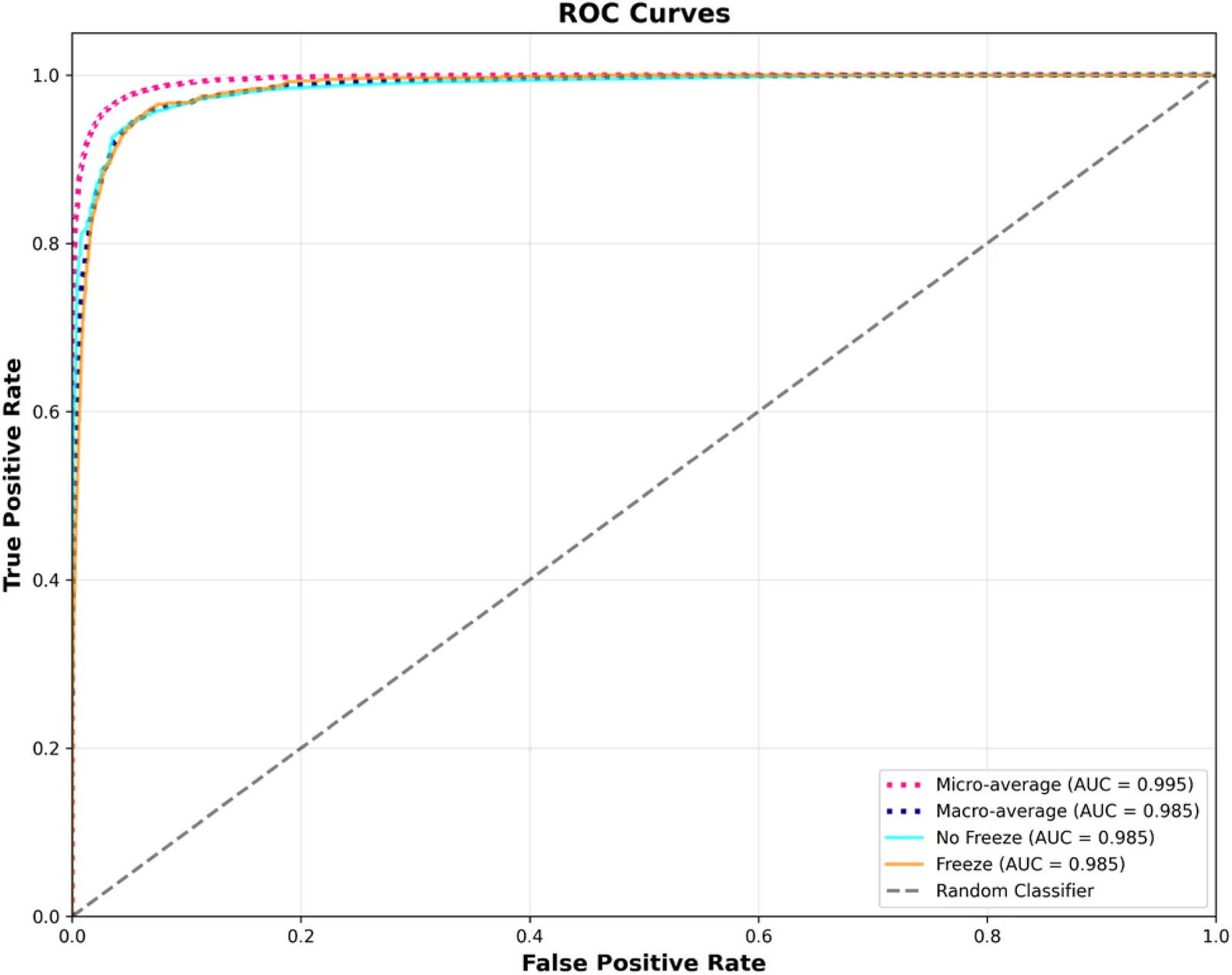
UniHAR results on the Daphnet dataset: (ROC) curves show the class-wise discrimination performance of the proposed model.

**Fig 14:**
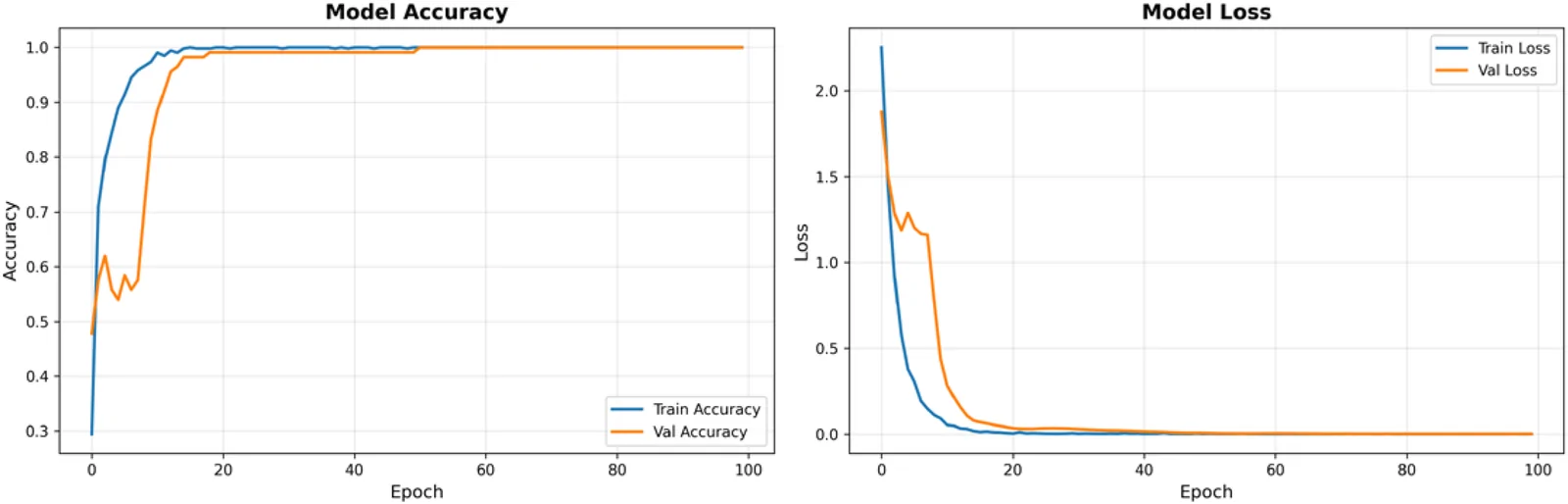
UniHAR results on the PAMAP2 dataset: training and validation curves illustrating the convergence behavior of the proposed UniHAR model.

**Fig 15:**
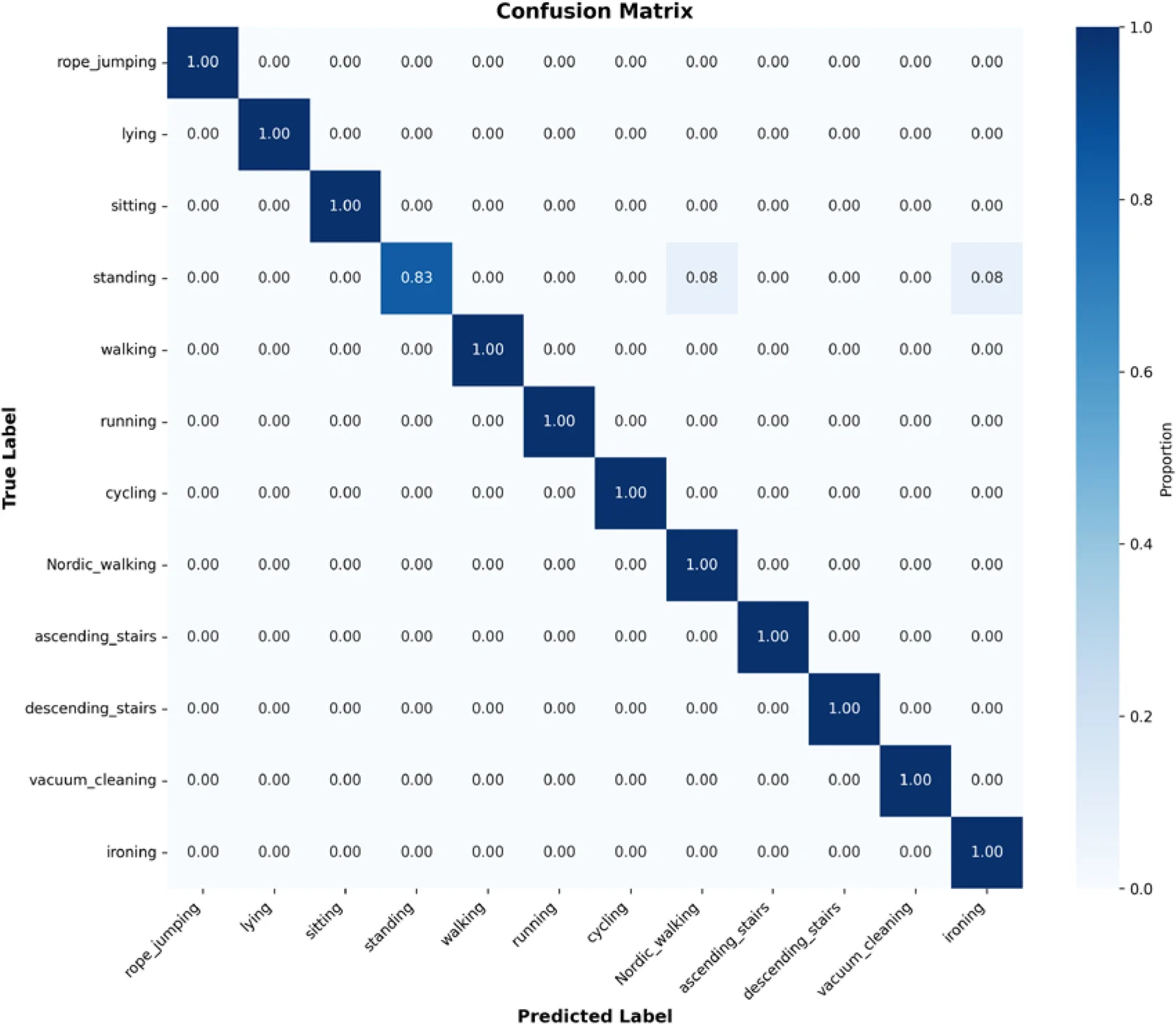
UniHAR results on the PAMAP2 dataset: confusion matrix shows the class-wise classification performance of the proposed model.

**UCI-HAR.** The training and validation curves Fig 5 of UCIHAR dataset show stable convergence and display a good generalization during training.

Our model, as shown in the confusion matrix Fig 6 successfully discriminates between conceptually similar activities.

Class separability is excellently confirmed by the ROC curves Fig 7 across all six activities. UniHAR achieve an accuracy of 96.35% and an F1-score of 96% in the UCI-HAR dataset.

**Opportunity.** UniHAR achieves an accuracy of 94.45% and an F1-score of 94%. The training and validation curves Fig 8 indicate stable convergence and effective generalization during training.

The confusion matrix Fig 9 shows improved discrimination between closely related activity categories, while the ROC curves Fig 10 further confirm strong class separability across all 18 mid-level classes.

**Daphnet.** UniHAR attains an accuracy of 96.44% and an F1-score of 96%. The training and validation curves Fig 11 indicate stable convergence and good generalization.

The confusion matrix Fig 12 demonstrates robust detection of the clinically critical “frozen” class under severe class imbalance, while the ROC curves Fig 13 show that all classes achieve an AUC greater than 0.95.

**PAMAP2.** UniHAR reaches an overall classification accuracy of 99.23% and an overall F1 score of 99%. All of these values are better than those reached by UniHAR’s baseline models. The training curve for UniHAR is shown in Fig 14 to be convergent.

The confusion matrix for UniHAR is shown in Fig 15 as well as being convergent, and shows that UniHAR was able to recognize each of the twelve classes with a good degree of accuracy.

Fig 16 confirms that UniHAR was able to produce good separations among the data points, since it has an area under the receiver operating characteristic curve greater than 0.985 for each class.

**Fig 16:**
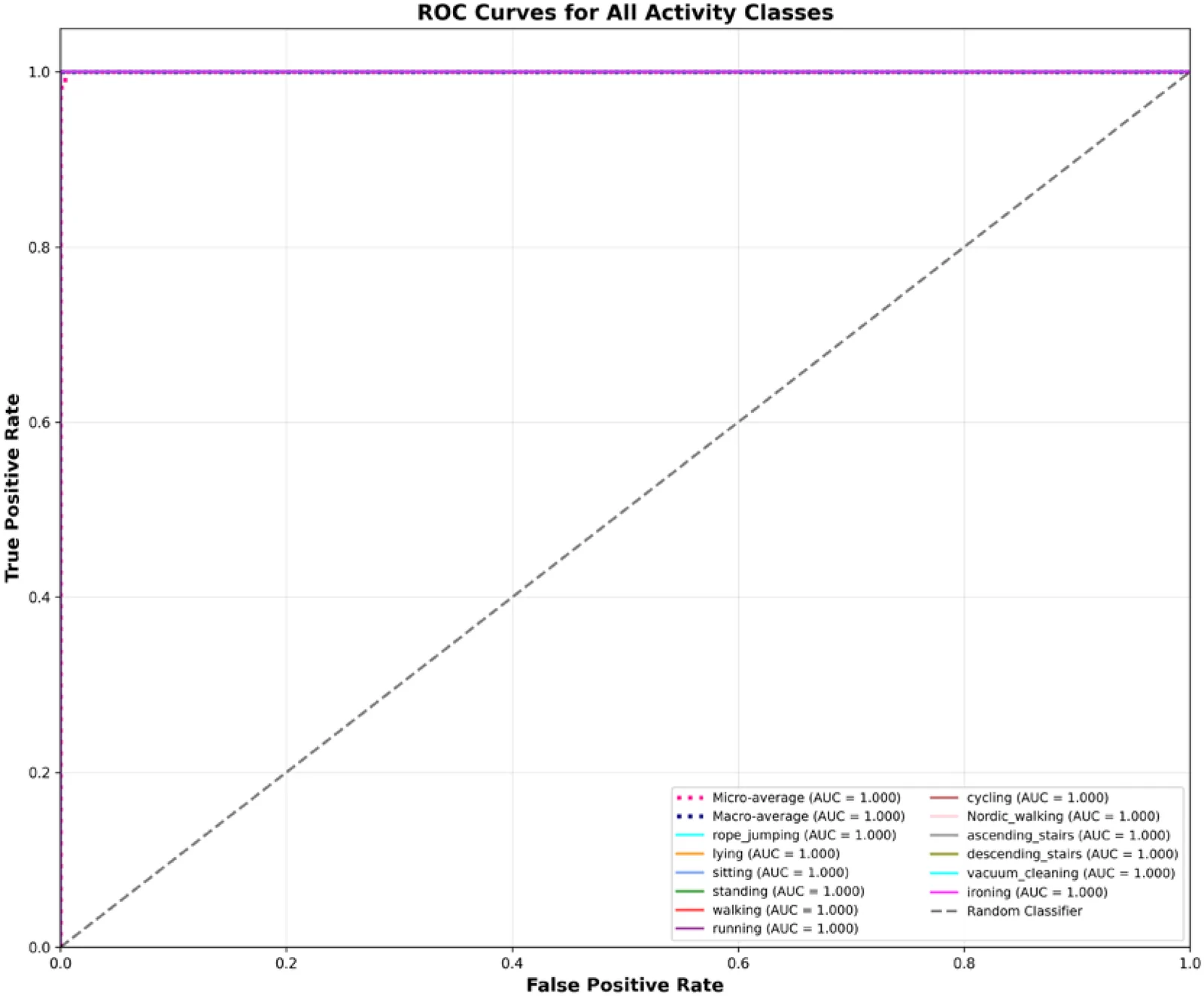
UniHAR results on the PAMAP2 dataset: (ROC) curves show the class-wise discrimination performance of the proposed model.

UniHAR also demonstrates practical inference efficiency, requiring approximately 5 ms per input window on a desktop CPU.

### 4.3 Zero-shot cross-dataset generalization results

The zero-shot cross-validation experiments evaluated UniHAR on six shared activities between UCI-HAR and PAMAP-2. No domain-independent samples were used for training, verification, normalization, hyperparameter estimation, and fine-tuning. The results, based on five independent source-domain training runs, are summarized in Table 12.

**Table 12:** Zero-shot cross-dataset generalization performance.

Source	Target	Input Method	Accuracy (%)	Macro-F1 (%)	Weighted-F1 (%)	Balanced Acc. (%)
UCI-HAR	PAMAP2	Common-channel selection	59.8	56.2	57.3	55.1
		Fixed padding	63.1	59.3	62.2	60.3
		Channel-mean aggregation	66.0	62.1	64.7	65.1
		**UCH**	**73.7**	**69.9**	**72.8**	**71.1**
PAMAP2	UCI-HAR	Common-channel selection	65.5	61.3	63.7	62.1
		Fixed padding	69.2	66.8	68.3	67.9
		Channel-mean aggregation	72.8	71.1	74.0	72.7
		**UCH**	**79.8**	**77.6**	**78.3**	**78.4**

For UCI-HAR→PAMAP2 transfer, From an accuracy of 73.7%, a macro-F1 score of 69.9%, and a balanced accuracy of 71.1%. the average descriptor grouped by the method of common descriptors gives an advantage over disabling additional channels and averaging their representations. Compared with common channel selection, constant padding and averaging, UCH improved 13.7, 10.6, and 7.8 percentage points, respectively.

For PAMAP2→UCI-HAR transfer, In the classification test, the unique cross-classifier performed remarkably, yielding an accuracy of 79.8% and a macro-F1 score of 77.6%. In this test, the unique cross-classifier outperformed the nearest competing technique, channel-mean aggregation, by a significant margin of 6.5 percentage points in macro-F1. thus indicating a greater tendency toward the 6→18 and 18→6 channel transitions.

Transfer from PAMAP2 to UCI-HAR yielded an F1 macro score 7.7 percentage points higher than the reverse. One explanation is that PAMAP2 contains measurements from the hand, chest, and ankle and thus provides a wider source-referenced spatial information base than the single-sensor UCI-HAR configuration.

#### 4.3.1 Source-to-target generalization gap.

The generalization gap was calculated as


$$\begin{array}{c} \Delta_{gen}=F1_{source}-F1_{target}. \end{array}$$ (37)

Compared to the different channel-handling strategies, the UCH method had the lowest relative generalization gap, with a reduction of 26.2 percentage points for UCI-HAR→PAMAP2 and 21.3 percentage points for PAMAP2→UCI-HAR. These results suggest that the UCH method more effectively transfers the source-based performance when transferring to a different channel configuration. Table 13 show the source-to-target generalization scores

**Table 13:** Source-domain and zero-shot target-domain macro-F1 scores.

Source	Target	Method	Source F1 (%)	Target F1 (%)	$\Delta_{gen}$
UCI-HAR	PAMAP2	Common-channel selection	93.3	56.2	37.1
		Fixed padding	93.9	59.3	34.6
		Channel-mean aggregation	95.7	62.1	33.6
		**UCH**	**96.1**	**69.9**	**26.2**
PAMAP2	UCI-HAR	Common-channel selection	93.9	61.3	32.6
		Fixed padding	97.1	66.8	30.3
		Channel-mean aggregation	96.9	71.1	25.8
		**UCH**	**98.9**	**77.6**	**21.3**

#### 4.3.2 Class-wise transfer performance.

Walking was expected to yield the best transfer performance, with F1 scores of 82.9% and 89.4% for UCI-HAR→PAMAP2 and PAMAP2→UCI-HAR, respectively. The main classification errors were expected between standing and sitting, and between climbing and descending stairs, as these two sets of actions had similar temporal characteristics and because they were sensitive to differences in positioning and orientation of the sensors. The standing posture was expected to be the most difficult, yielding F1 scores of 59.4% and 69.5% in the two transfer directions. Table 14 show the class-wise zero shot f1 scores.

**Table 14:** Class-wise zero-shot F1 scores for UCH.

Activity	UCI-HAR→PAMAP2	PAMAP2→UCI-HAR
Walking	82.9	89.4
Walking upstairs	68.6	75.7
Walking downstairs	66.2	73.6
Sitting	62.3	71.3
Standing	59.4	69.5
Lying	80.0	86.1
**Macro average**	**69.9**	**77.6**

#### 4.3.3 Representation alignment.

The channel homogenization reduced the MMD from 0.437 to 0.215, while the channel aggregation reduced it from 0.437 to 0.322, which is less efficient than the learned channel grouping. Table 15 shows the discrepancy averaged across the two transfer directions.

**Table 15:** Source–target representation discrepancy averaged across the two transfer directions. Lower MMD values indicate better alignment.

Representation method	MMD
Before harmonization	0.437
Channel-mean aggregation	0.322
**UCH**	**0.215**

#### 4.3.4 Robustness to channel removal.

At a channel-removal ratio of 25%, UCH lost 4.2 percentage points, whereas fixed padding and channel-mean aggregation lost 6.7 and 4.9 percentage points, respectively. When half of the sensor channels were unavailable, UCH retained a macro-F1 score of 63.2%, corresponding to a reduction of 10.6 percentage points from its complete-channel performance. Table 16 shows the target-domain macro-F1, averaged across both transfer directions, under random channel removal.

**Table 16:** Target-domain macro-F1 averaged across both transfer directions under random channel removal.

Removed channels	Fixed padding	Mean aggregation	UCH
0%	63.1	66.6	**73.8**
10%	60.8	65.1	**71.8**
25%	56.4	61.7	**69.6**
50%	47.9	53.8	**63.2**
75%	35.6	41.7	**51.4**

#### 4.3.5 UCH internal ablation.

Uniform channel-mean pooling produced a target-domain macro-F1 score of 66.60%. Incorporating only statistical descriptors increased macro-F1 to 70.05%, while using only spectral descriptors resulted in 69.35%. Random channel grouping achieved 68.40%, showing that independent channel processing alone would not explain the complete UCH improvement.

Full UCH achieved the highest target-domain macro-F1 of 73.75% and the lowest MMD of 0.215. Compared with mean pooling, it introduced approximately 0.04 million additional parameters and 0.34 ms of additional inference latency, while remaining within the sub-5 ms inference range reported for the original UniHAR architecture. Table 17 shows the internal ablation results.

**Table 17:** Internal ablation results averaged across the two cross-dataset transfer directions.

Variant	Source F1 (%)	Target F1 (%)	Balanced Acc. (%)	MMD	Parameters (M)	Latency (ms)
Channel-mean pooling	96.30	66.60	68.90	0.322	0.80	3.58
UCH-statistics	96.80	70.05	70.75	0.257	0.83	3.76
UCH-spectrum	96.55	69.35	70.05	0.274	0.83	3.81
UCH-random groups	96.30	68.40	69.10	0.298	0.82	3.73
**Full UCH**	**97.50**	**73.75**	**74.75**	**0.215**	0.84	3.92

#### 4.3.6 Effect of the number of latent groups.

A single latent group effectively behaved as weighted global channel pooling and achieved a macro-F1 score of 66.60%. Increasing the group count to two improved macro-F1 to 71.00%, while four groups achieved the best performance of 73.75%. Increasing the number of groups to eight slightly reduced performance to 72.40%, suggesting that excessive grouping fragments the available channel information. Table 18 shows the effect of the latent group count on target-domain performance.

**Table 18:** Effect of the latent group count on target-domain performance.

M	Macro-F1 (%)	MMD	Parameters (M)
1	66.60	0.322	0.80
2	71.00	0.252	0.82
4	**73.75**	**0.215**	0.84
8	72.40	0.226	0.88

#### 4.3.7 Statistical significance.

Using direction-averaged results from five matched random seeds, full UCH improved target-domain macro-F1 by 7.15 percentage points over channel-mean aggregation. A one-sided exact Wilcoxon signed-rank test and a bootstrap 95% confidence interval were used to assess the statistical significance and uncertainty of this improvement.

## 5 Discussion

Errors were not random but systematic. On UCI-HAR, 26% of standing samples were misclassified as sitting (Fig 6). This is because of the very low variability of both postures in the IMU data; the coarse-scale AMSPE stream is unable to resolve the ambiguity. On the Opportunity, errors are confined to the fine-grained and low-frequency m-values (e.g., drawer-open/close, Fig 9). As noted in Table 19, the FTCA approach cost 12.1 points of accuracy on the Opportunity (94.45%→82.34%) compared to 4.87 and 2.34 points on UCI-HAR and PAMAP2, which is not tested here since all four test datasets are densely recorded. Indicating that channel-dependency modeling, not multi-scale temporal modeling, is disproportionately load-bearing on high-channel-count datasets (*C* = 113), and that the model’s robustness margin there is narrower than headline accuracy suggests.

**Table 19:** Accuracy (%) of component-wise ablation variants across datasets.

Configuration	UCI-HAR	Opp.	Daph.	PAMAP2
**Full UniHAR**	**96.35**	**94.45**	**96.44**	**99.23**
w/o UCH	93.89	87.62	92.31	95.47
w/o AMSPE	92.24	85.89	91.78	97.71
w/o SAFCA	92.12	91.58	90.89	97.85
w/o ISIE	96.21	92.87	96.38	98.18
w/o FTCA	91.48	82.34	92.12	96.89
w/o Fourier mixing	94.17	93.24	91.56	98.94

Architecturally, UCH’s channel-count generalization relies on a soft upper bound: channels beyond $C_{\max}=113$ are zero-padded and suppressed only insofar as the group-assignment scorer learns near-zero weights for them (Section 3.2.4), which is an approximation rather than a guarantee and is untested here since all four benchmarks satisfy C≤113. ISIE’s coarse→medium→fine message-passing direction (Section 3.4.2) is fixed a priori rather than learned or data-adaptive; a bidirectional scheme was proposed but not implemented, and this rigidity may partly explain the static-posture errors above, where coarse-scale context should dominate but cannot be reweighted per activity.

On efficiency, UniHAR’s inference latency (2.74 ms on UCI-HAR, batch size one, Table 7) exceeds eight of nine baselines, including CNN (0.72 ms) and DKInception (1.13 ms), and its parameter count (0.782M) exceeds several lightweight baselines by 2–9×; the efficiency claim is therefore a favorable accuracy–cost trade-off rather than outright superiority.

On generalization, the zero-shot cross-dataset experiments (Section 4.3) show UCH narrows but does not close the source-to-target gap: target-domain macro-F1 remains 22–26 points below source-domain performance (Table 13), and this evaluation covers only a six-class harmonized subset shared between UCI-HAR and PAMAP2 rather than either dataset’s full taxonomy. All four evaluation datasets are wearable-IMU-based; extension to other HAR modalities (WiFi CSI, mmWave radar, vision-based skeletons) is untested, and channel harmonization across modalities with different physical semantics may require descriptor design beyond the statistical/spectral features used in UCH (Section 3.2.1).

The results of these experiments suggest several concrete extensions. Adaptive or learned channel-count bounds could replace the static zero-padding. Similarly, a bi-directional AMSPE scheme could refine the static-posture error.

### 5.1 Ablation study

To determine how much the performance of the UniHAR system depends on the various constituents of the system, a series of systematic ablation experiments were carried out on the system, using the four available data. These studies showed a different impact on the different combinations of sensors, each of which was used in a different way for each data set. The ablation results of the individual components of the UniHAR system, tested against all data sets, are shown in Table 19. This table demonstrates how the removal of each individual module affects the overall performance of the UniHAR system.

## 6 Conclusion

In this study, a hierarchical multi-scale Transformer, UniHAR, is proposed for sensor-based human activity recognition. The model is suitable for edge deployment to address the heterogeneous sensor configurations and multi-scale temporal modeling. Integration of multiple modules, including Adaptive Multi-Scale Patch Embedding, Universal Channel Harmonization, Factorized Temporal–Channel Attention and Fourier-based cross-scale attention. On four benchmark datasets our model achieves state-of-the-art performance with only sub-5ms inference latency on a desktop CPU and a lightweight architecture. Complementary contribution of each module is proved by an ablation study. In future work, our focus will be on imbalance-aware learning, adaptive module selection, and cross-dataset generalization to further improve the health care assistive applications.
